# Small Extracellular Vesicle Release Following Electrical Pulse Stimulation of C2C12 Myotubes: Effects on microRNA Cargo and Myoblast Migration and Differentiation

**DOI:** 10.3390/ijms27104320

**Published:** 2026-05-12

**Authors:** John S. Hingle, Rhys S. McColl, Ivan J. Vechetti, Kathryn H. Myburgh

**Affiliations:** 1 Department of Physiological Sciences, Stellenbosch University, Stellenbosch 7602, South Africa; 22761039@sun.ac.za (J.S.H.); rhysmccoll1@gmail.com (R.S.M.); 2 Department of Nutrition and Health Sciences, College of Education and Human Sciences, University of Nebraska-Lincoln, Lincoln, NE 68583, USA; ivechetti@unl.edu

**Keywords:** extracellular vesicles, C2C12 myotubes, electrical pulse stimulation, micro-RNA cargo

## Abstract

The skeletal muscle (SkM) secretome has been widely studied since the establishment of its endocrine function. Extracellular vesicles (EVs) are the most recently identified elements of the SkM secretome. These nano-sized lipid-bound vesicles carry molecular cargo and function as a means of intercellular communication. The effect of exercise on SkM EV micro-RNA cargo (miRNAs) remains a challenge to elucidate. Electrical pulse stimulation (EPS) was applied to C2C12 myotubes at high (30 Hz) and low (2 Hz) frequencies. EVs released during 10 h of stimulation were isolated and characterized and used to treat myoblasts. Their miRNA cargo was sequenced. EVs were used to treat myoblasts (2.19 × 10^8^ EVs per mL) to determine the effects on myoblast migration and differentiation. Sequencing revealed over 300 known miRNAs packaged into myotube EVs. Many were differentially expressed after EPS, either positively or negatively. Muscle-important miRNAs were present (miR-206 was 4.8-fold more prevalent than any other miRNA). EV treatments improved myoblast migration and differentiation without a frequency-specific influence. Gene Ontology analysis based on differentially expressed miRNAs between control and EPS-EVs indicates an effect of EPS frequency on muscle EV signaling.

## 1. Introduction

Skeletal muscle is known to be a highly metabolic organ, which functions to generate force [1]. In the last decade, it has been established that skeletal muscle functions as an endocrine organ, releasing hormones, cytokines, and growth factors, which are proposed to be the mechanisms for at least some of the beneficial effects of exercise on other organs [2]. Exercise has been shown to lead to health benefits, including positive effects on metabolism and insulin sensitivity [3], neurological health [4], cardiac health [5], and many more. More recently extracellular vesicles (EVs) have been proposed as mediators of systemic adaptations to exercise [6] and are considered a part of the muscle secretome [7]. However, studying the muscle secretome during exercise is complex and so using in vitro models can simplify the approach and hone in on specific factors released only by skeletal muscle.

EVs are nano-sized, are enclosed by a lipid bilayer, and contain various cytosolic components from the parent cells that release them [8]. EVs are released by all cell types and are involved in both physiological and pathophysiological processes [9]. EV size, biogenesis, and mode of release differ between the different types of EVs [8]. EVs are further subdivided according to size, though it should be taken into consideration that EV size is a continuum. Complications in EV nomenclature and methodology have been standardized according to the MISEV guidelines [10], which have been followed in conducting this EV study. Small EVs, in the size range of 50 to 200 nm, are the focus of this research study. They act as a means of intercellular communication and carry molecular cargo, the composition of which is often distinct from that of the parent cell. The main cargo of interest in small EV research is micro-RNAs (miRNAs) and their selective packaging into EVs.

EV isolations from various cell types have been discovered to contain distinct populations of RNA, both coding and short non-coding RNA [11,12]. Cellular RNA is enriched in larger full-length coding RNA species, while EVs are enriched in shorter RNA species including microRNA (miRNA), snoRNA, snRNA, and piRNA [13,14]. This has proven to be the case for the cargo of EVs released from C2C12 cells [15]. Not all miRNAs in muscle cells are packaged into EVs, and there are certain miRNAs that are found in EVs and not in detectable amounts in the parent cell [16], indicating a mechanism responsible for the selective packaging of miRNA into muscle EVs. MiRNA acts by degrading mRNA or interfering with mRNA–ribosome binding, resulting in the prevention of protein translation [17]. It is important to note that the regulation of gene transcription by miRNA is complex. A single miRNA can target multiple mRNAs, and a certain mRNA can be targeted by multiple miRNAs [18,19]. Therefore, multiple miRNAs play a collective role in altering protein translation.

The high energy demand of working muscle disturbs homeostasis and increases the work of these organs, setting up adaptive effects [20]. One such adaptive effect in muscle in response to exercise is the change in levels of muscle-important miRNAs, termed myomiRs [21,22,23]. These myomiRs, such as miR-206, 133a/b and miR-1, are expressed 20-fold higher in skeletal muscle than in other tissues and are involved in myogenesis and are potentially upregulated to induce adaptive changes in response to exercise [24,25]. Directly post-exercise, there is an increase in circulating free miRNA [26,27], as well as an increase in circulating EVs [28,29]. An important question remains: what proportion of these increased miRNA and EV levels are specific to muscle and released in response to exercise? This issue is complicated by the fact that plasma contains EVs from many sources including platelets and leukocytes [30]. An in vitro exercise model eliminates confounding EV sources and can focus on the effect of myotube contraction on the miRNA packaging of their EVs. Muscle contraction in vivo is brought about by motor neuron activation [31]. To mimic this, electrical pulse stimulation (EPS) is applied to myotubes in vitro. Various stimulation parameters can be adjusted, such as the pulse duration, voltage (V), and frequency of stimulation (Hz). Low Hz stimulation causes individual twitch contractions, and high Hz stimulation can result in fused tetanic contractions [32,33]. EPS has been shown to result in accelerated sarcomere assembly in C2C12 myotubes [34,35,36]. Other researchers have subsequently used EPS to study IL-6 release [37], Ca^2+^ transients [35], GLUT4 translocation [38], and the activation of pathways linked to exercise, including AMPK and mTOR [39,40].

This study utilized low Hz and high Hz EPS to simulate in vitro exercise bringing about twitch and tetanic contractions in C2C12 myotubes. EVs from the no-EPS control and from contracting myotubes were characterized, and their miRNA cargo was assessed. The miRNA sequencing of EV cargo and treatment of EPS-naïve myoblasts with these EVs are novel aspects of the current study. We also investigated myoblast migration and differentiation in response to treatment with EVs collected in response to EPS. We hypothesized that applying EPS to myotubes would alter EV release and the packaging of miRNA into EVs released into culture media.

## 2. Results

### 2.1. Model Development

**Figure 1:**
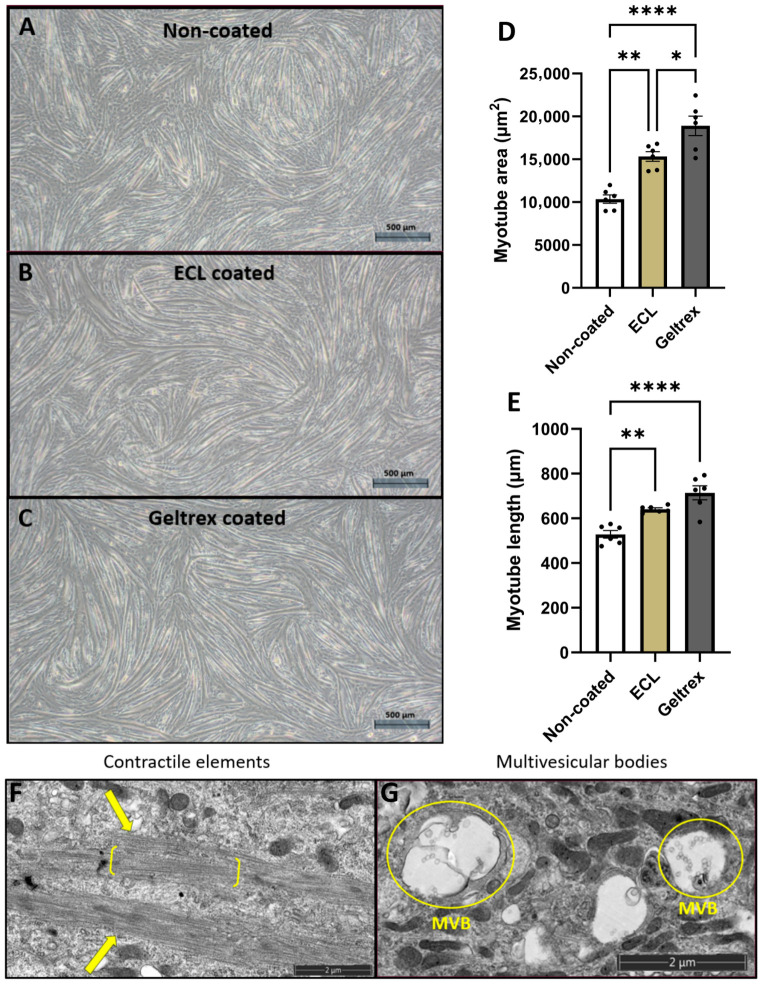
Myotube model development. (**A**) Myotubes grown in non-coated culture plates. (**B**) Myotubes grown in ECL-coated plates. (**C**) Myotubes grown in Geltrex-coated plates. All images were taken on day 5 of differentiation at 40× magnification. Scale bar: (**A**–**C**)—500 µm. (**D**) Average area of myotubes grown on different coatings. (**E**) Average length of myotubes grown on different coatings. Myotubes grown on ECL and on Geltrex were significantly larger and longer than those grown in non-coated culture plates. Data expressed as the mean ± SEM. Significant differences were determined using a one-way ANOVA with a Tukey post-hoc test. * = *p* < 0.05; ** = *p* < 0.01; **** = *p* < 0.0001. N = 6. Electron micrographs showing actin filament organization (arrows) and early sarcomere between brackets (**F**) and multivesicular bodies (**G**) in day-5 myotubes. Scale bar: (**F**,**G**)—2 µm.

In this project, it was imperative to optimize myotube formation to enable a viable in vitro exercise model. To achieve this, two different culture plate coatings (ECL (entactin-collagen IV-laminin) and Geltrex) were used to promote better myoblast alignment and fusion. Both ECL and Geltrex resulted in the formation of larger (ECL: *p* < 0.01; Geltrex; *p* < 0.0001) and longer (ECL: *p* < 0.01; Geltrex; *p* < 0.0001) myotubes compared to myotubes that formed on non-coated plates (Figure 1). Myotubes grown on Geltrex had a greater average area compared to ECL-grown myotubes (*p* < 0.05), though no statistical difference was found in the length of the myotubes. With the Geltrex coating, there were also fewer fields of view with unfused myoblasts (Figure 1).

Electron micrographs revealed what looked like actin filaments aligning after 5 days under differentiation conditions (Figure 1F). Previously, Rabieh et al. [41] showed evidence of fully formed sarcomeres in primary myotubes after 10 days. Also visible here were structures resembling vesicles. Some containing smaller vesicles were clearly seen and may represent multivesicular bodies (Figure 1G).

### 2.2. Extracellular Vesicle Characterization

**Figure 2:**
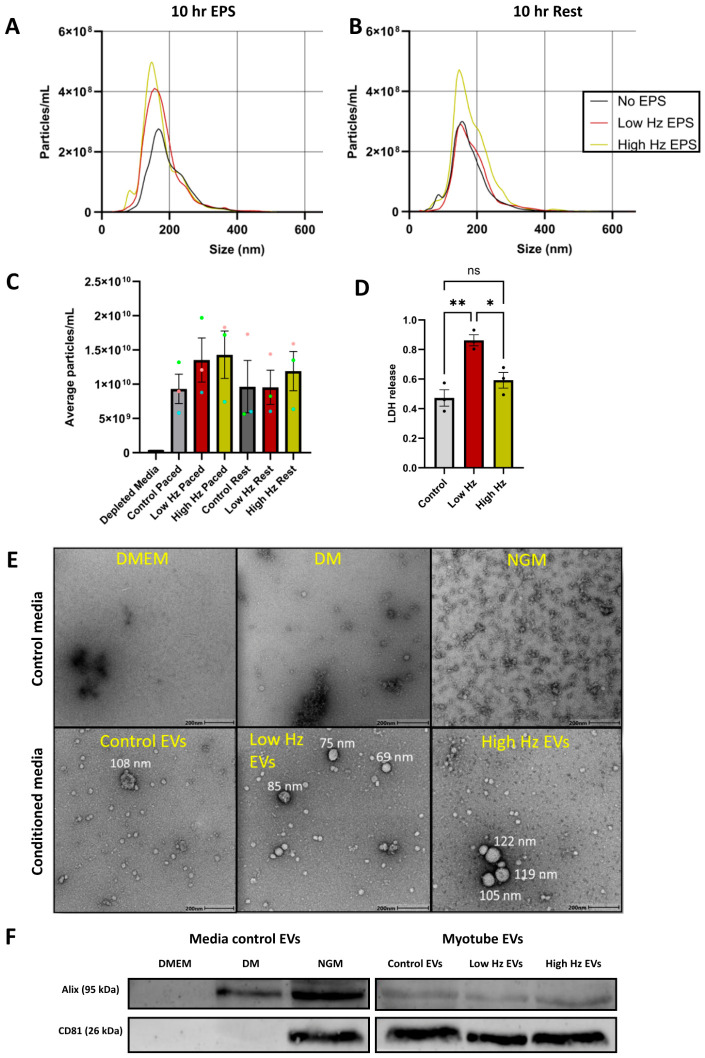
Characterization of extracellular vesicles released in response to EPS. NTA was performed on resuspended EV pellets. Each EV pellet was isolated from the collective conditioned media of three 6-well plates. Concentration in particles/mL of EVs collected directly after 10 h of EPS (**A**) and after a subsequent 10 h of rest (**B**). (**C**) Average concentration of myotube EV pellets. (**D**) Lactate dehydrogenase release into cell culture media by myotubes in response to EPS. Data expressed as the mean ± SEM. * = *p* < 0.05; ** = *p* < 0.01. N = 3. (**E**) TEM images of resuspended EV pellets were taken from three media conditions prior to cell seeding. DMEM, depleted media (DM), and normal growth media (NGM). This was repeated for conditioned media EV pellets from non-stimulated control myotubes, low-Hz-stimulated myotubes, and high-Hz -stimulated myotubes. Scale bar—200 nm. (**F**) Western blotting was performed on resuspended EV pellets of the three media controls, as well as from resuspended EV pellets of the three myotube groups.

Nanoparticle tracking analysis (NTA) was used to determine the concentration (particles per mL) and size of particles (diameter in nm) in an EV isolation. Size distribution graphs (Figure 2A,B) show a single peak indicative of a single population of EVs. NTA results from resuspended EV pellets showed no differences in particles per mL or in mean and mode sizes, in response to the 10 h stimulation protocol (Figure 2). There was little variability in the mean size and mode size. This may have been expected as the EVs were collected from a single cell type. NTA results from EVs collected from the same myotubes after 10 h post-stimulation rest also revealed no differences between the EV number and size.

Transmission electron microscopy was used to visualize individual EVs at high resolution. Images were used to qualitatively assess the size and shape of particles. For both media controls and myotube EV groups, EVs were isolated with differential ultracentrifugation, and the EV pellet was resuspended for use in transmission electron microscopy (TEM) preparation. No EVs were present in the TEM micrographs (Figure 2E) of the DMEM media control, as would be expected. The micrograph of depleted media showed very few particles, of sub 50 nm in size, indicating the successful depletion of EVs. In contrast, many small particles were present in the normal growth media, although the micrograph indicates that these were still in the sub-50 nm size range. Micrographs of resuspended EV pellets from all three myotube conditioned media groups indicate the presence of larger exosome-sized particles, as well as smaller sub-50 nm particles. Western blotting was performed on resuspended EV pellets of control media and myotube EV groups to confirm the presence of pan-EV markers specific to exosomes (Alix and CD81) (Figure 2F). In the control media, DMEM showed no bands present. In the depleted media group, there was a faint Alix band present, though no bands were present for CD81. Normal growth media had a definitive band present for both exosome markers as expected. In the myotube-derived EVs there were fainter bands present for Alix and strong bands present for CD81, with no apparent differences among the three EV groups. CD81 was shown to be the best marker for the presence of EVs, possibly due to it being EV-membrane-bound as opposed to Alix, which is involved in the packaging of exosomes.

### 2.3. Extracellular Vesicle miRNA Cargo Analysis

To gain insight into the EV cargo of the two myotube stimulation groups compared to the control no stimulation group, miRNA sequencing was performed. Four EV pellets from each of the three myotube groups (N = 12) were pooled, resulting in a 1.6 mL suspension. In addition, a depleted media control sample was pelleted. This sample was media made up with EV-depleted FBS. This media contained very few EVs; nonetheless, these were pelleted and resuspended. A portion was sent for sequencing. The remainder was aliquoted to be used in EV treatments at a later stage of the project. The results from the sequencing reports included the RNA concentration of EV samples, EV small RNA quality control and composition, and known miRNAs sequenced.

Small RNA sequencing results determined that there were 324 miRNAs present inside the myotube-derived EVs. Not all miRNAs were found in high quantities, with 124 miRNAs having 100 reads per million (RPM) or more and the top 52 miRNA being over 1000 RPM. Data was normalized by converting raw read counts into trimmed mean of M-values (TMM) for further analysis. The depleted media sample contained a much lower total RNA concentration compared to the myotube EV groups, indicating that most of the RNA in the myotube EV groups is packaged inside EVs released by the myotubes themselves and is not free-floating. Small RNA analysis results of the pellet gained from depleted control media also contained very few miRNAs, compared to myotube EV pellets (view Supplementary file S1 to access miRNA sequencing reports and full EV miRNA cargo list).

Table 1 presents the top 30 most abundant miRNAs ranked according to control non-stimulated EV miRNA TMM levels. The sequencing results revealed that miR-206-3p was by far the most prevalent in the myotube-derived EVs in all three groups, more than 4-fold higher than any other miRNA. miRNAs ranked 2, 3, and 4 were between 40,000 and 72,000 in the control myotubes. Of the miRNAs ranked 4–10, five were in the let-7 family. These results were derived from pooled samples. Although the pellets were derived from 12 × 6-well plates, with each of the twelve sets incubated in different weeks, differential miRNA abundance at the individual miRNA level can be seen as exploratory. Nevertheless, Table 1 presents fold changes for the two EPS groups compared to the no-stimulation control. Where trends appeared, the two EPS groups behaved in a similar fashion. The only striking results were for miR-486a-5p, and miR-486b-5p and miR-615-3p were reduced by approximately 50%.

Table 2 represents known myomiRs that were present in the myotube EVs. For miR-206 and miR-133a and b, the fold difference appeared to be higher with EPS, whereas for miR-1a and miR-486a and b, the fold changes were less than 1.

### 2.4. Gene Ontology Enrichment of Targeted Myogenic Pathways

To analyze the systemic impact of the altered EV cargo as a result of electrostimulation, we mapped differentially expressed miRNAs (Log_2_FC > 1 or < −1) to their mRNA targets using the multiMiR Bioconductor package. To move beyond low-confidence predictions, these targets were identified using a multi-pronged approach aggregating experimentally validated interactions (miRTarBase and TarBase) and high-confidence predicted targets (miRDB) via the multiMiR package. The miRTarBase and TarBase databases consist exclusively of experimentally validated miRNA–target interactions (supported by luciferase reporter assays, Western blots, and CLIP-Seq), ensuring that the downstream analysis reflects established empirical data. The results were stratified into “Suppressed” states (processes and hub gene targets of upregulated miRNAs) and “Activated/Derepressed” states (processes and hub gene targets of downregulated miRNAs) to map the directional regulatory pressure applied by the EV cargo based on myogenic biological processes. The results from this predictive analysis are presented in six dot plots and a heatmap.

**Figure 3:**
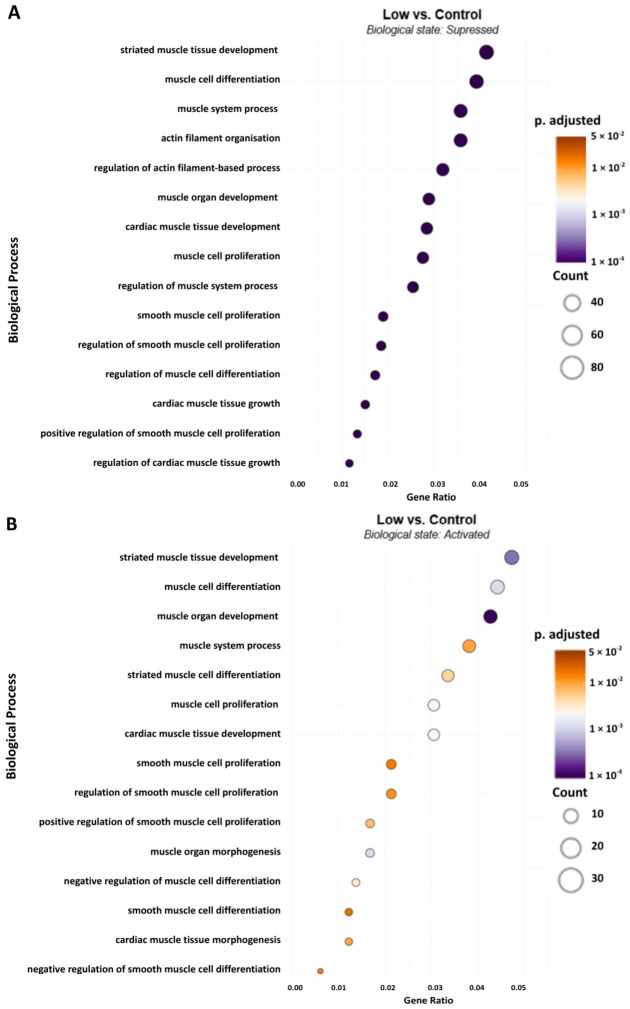
Dot plot representing the functional enrichment of biological process terms associated with muscle regeneration and remodeling across experimental groups low vs. control for both suppressed (**A**) (miRNA-targeted) and activated (**B**) (miRNA-derepressed) states. The x-axis represents the gene ratio (standardized to 0.06), defined as the proportion of differentially targeted genes within a specific GO term. Dot size corresponds to the absolute gene count, while the color gradient indicates the statistical significance (Benjamini–Hochberg adjusted *p*-value).

There was a robust miRNA response across the experimental groups. The low Hz vs. control group exhibited a distinct profile, with 42 upregulated and 55 downregulated miRNAs. The biological processes with the highest number of genes relative to the total number of genes (gene ratio) downregulated were those involving developmental processes defined as muscle tissue or organ development (Figure 3A). It is noteworthy that low Hz stimulation suppressed processes defined as being involved in smooth or cardiac muscle. Two biological processes that were downregulated at a significant level were actin filament organization and regulation. A determination of relevant hub genes is provided below in the heatmap. Biological states activated in the low Hz group overlapped to some extent with those suppressed, due to the relatively larger proportion of genes represented by these processes. These predictions suggest a multifaceted response.

**Figure 4:**
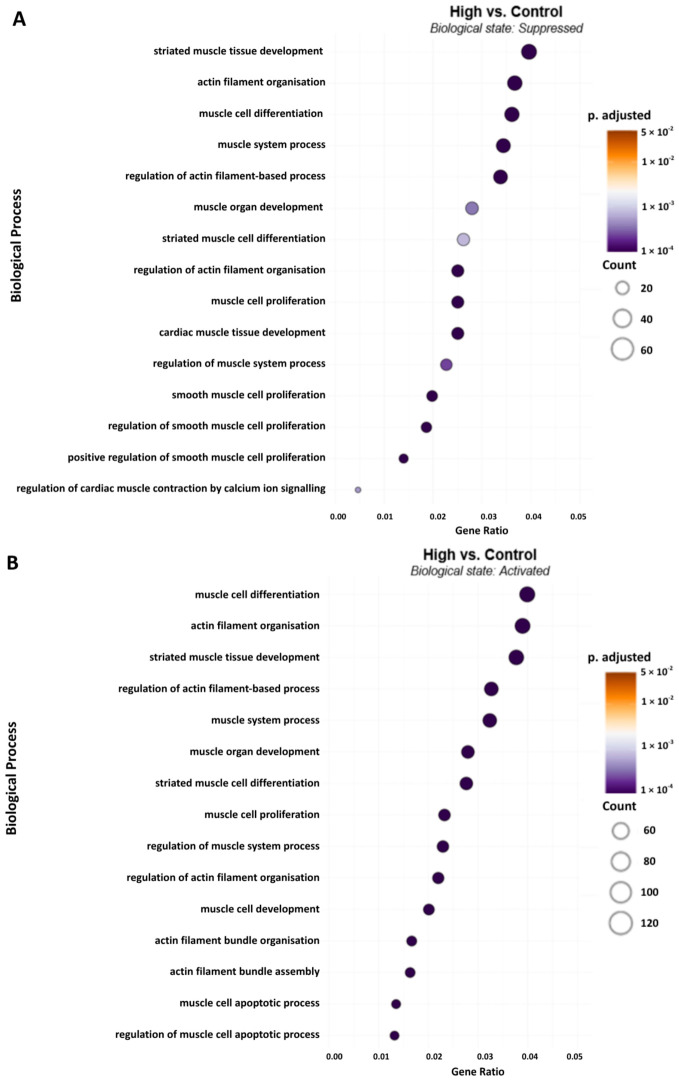
Dot plot representing the functional enrichment of biological process terms associated with muscle regeneration and remodeling across experimental groups high vs. control for both suppressed (**A**) (miRNA-targeted) and activated (**B**) (miRNA-derepressed) states. The x-axis represents the gene ratio (standardized to 0.06), defined as the proportion of differentially targeted genes within a specific GO term. Dot size corresponds to the absolute gene count, while the color gradient indicates the statistical significance (Benjamini–Hochberg adjusted *p*-value).

In the high Hz vs. control group, 59 miRNAs were significantly upregulated, while 68 were downregulated. Figure 4 indicates the same overlap between processes suppressed (Panel A) and activated (Panel B). Biological processes influenced by high Hz stimulation with a lower gene ratio had less overlap between suppression and activation. High Hz stimulation clearly suppressed five biological processes involved in cardiac and smooth muscle regulation. Conversely, apoptotic processes were only activated but with few genes involved (the last two processes listed in Figure 4B).

An analysis of the processes suppressed by upregulated miRNAs in the high Hz group compared to the control revealed a focus on striated muscle tissue development (p.adj = 8.99 × 10^−7^) and actin filament organization (p.adj = 8.68 × 10^−6^). These processes involve a cluster of high-connectivity mRNA targets, including *Mef2c* (Pathway Count: 62), *Camk2d* (52), and *Igf1* (50) (see also heatmap, below). Pathways activated (derepressed) in the high Hz group compared to the control were dominated by muscle cell differentiation (p.adj = 5.14 × 10^−14^) and muscle organ morphogenesis (p.adj = 2.83 × 10^−1^).

A direct comparison of high Hz vs. low Hz groups identified 51 miRNAs specifically differentially upregulated in the high Hz group and 62 downregulated.

**Figure 5:**
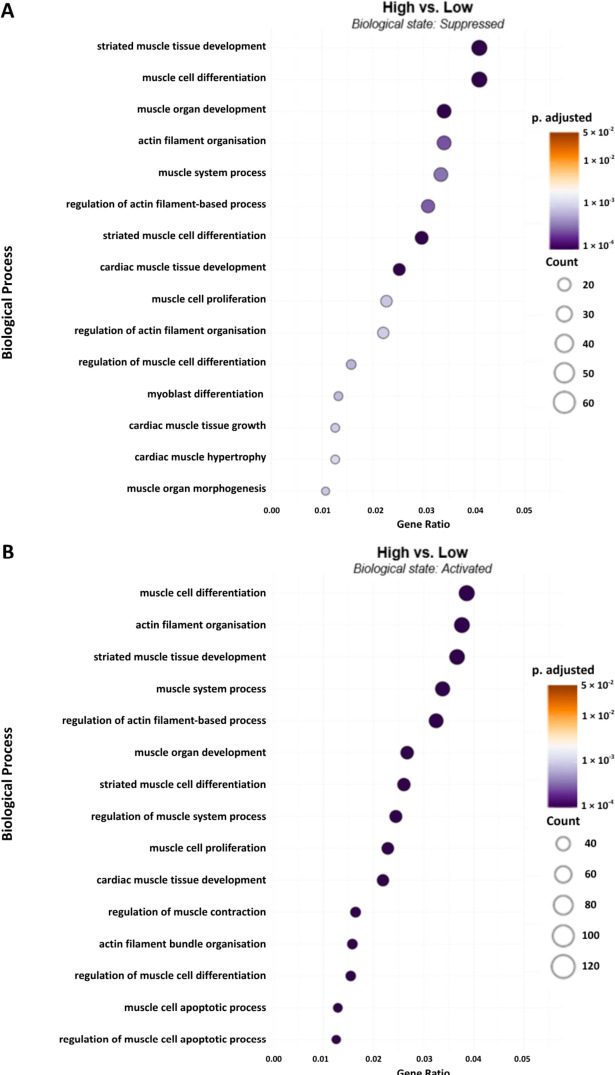
Dot plot representing the functional enrichment of biological process terms associated with muscle regeneration and remodeling across experimental groups low vs. high for both suppressed (**A**) (miRNA-targeted) and activated (**B**) (miRNA-derepressed) states. The x-axis represents the gene ratio (standardized to 0.06), defined as the proportion of differentially targeted genes within a specific GO term. Dot size corresponds to the absolute gene count, while the color gradient indicates the statistical significance (Benjamini–Hochberg adjusted *p*-value).

Figure 5A represents the processes that were more suppressed in high Hz vs. low Hz, and the dominant process was striated muscle development, whereas the process most activated in high Hz was muscle cell differentiation. Also suppressed in high relative to low Hz were “muscle system processes,” which include aspects of muscle contraction (e.g., activation, excitation-contraction coupling, and relaxation).

**Figure 6:**
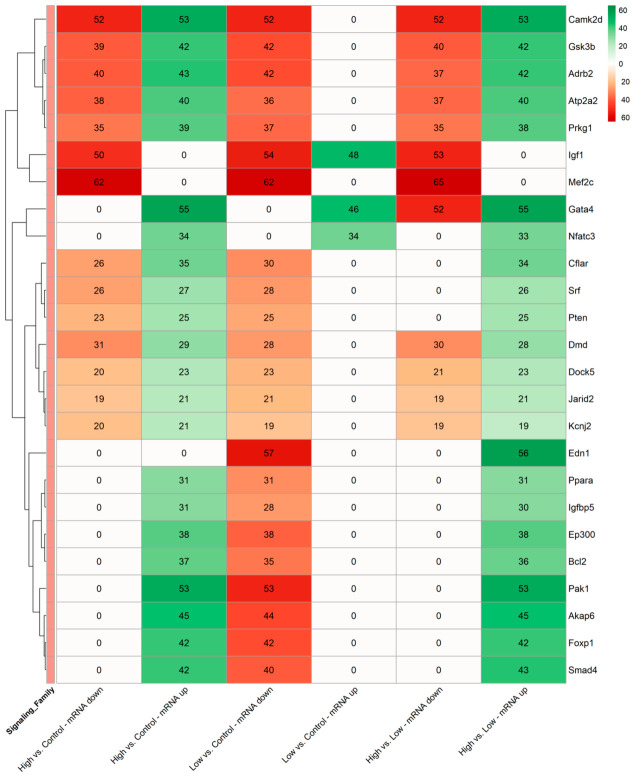
Annotated Heatmap illustrating the predicted functional connectivity of the top 25 mRNA “Hub” targets across all experimental conditions. Columns stratify results into “mRNA down” (hubs inhibited by upregulated EV-miRNAs) and “mRNA up” (hubs derepressed by downregulated EV-miRNAs). Red: hub mRNA suppression. Green: hub mRNA derepression. The red/green scale represents regulatory intensity. Cell values: a connectivity score (total number of significant muscle-specific GO pathways regulated by the hub genes). A connectivity score of 0 = no predicted regulatory pressure from the EV-miRNAs on that specific gene in that specific comparison. Vertical annotation sidebar (right): categorizes each hub by a gene that is primary in its signaling family. Vertical annotation (left): hierarchical clustering of rows to reveal functional modules of co-regulated genes.

Given the broad categorization of the biological processes identified above, a heatmap was generated to identify hub genes (Figure 6).

On the right side of the heatmap (Figure 6), important “hub genes” identified by the GO pathway analysis are listed with connectivity scores indicated in the rows. The connectivity scores indicate the absolute number of discrete, GO-identified, muscle-related pathways in which these mRNAs are involved. The use of a divergent red/green color scheme in Figure 6 provides a visual representation of this signaling polarity. Red gradients (mRNA down) identify the “inhibitory modulation” of the EV miRNA cargo, whereas green gradients (mRNA up) highlight the “promotional potential” via the removal of constitutive miRNA-mediated brakes (derepression).

High Hz stimulation consistently increased the number of pathways connected to hub genes that indicate upregulation. This can be seen when comparing high Hz to control and to how Hz. The hub genes with the highest connectivity scores were *Gata4*, *Pak1*, and *Camk2d* when comparing high Hz to control. These three also emerged when comparing high Hz to low Hz, but *Edn1* also emerged with a very high connectivity score. Although fewer hub genes were identified as downregulated with high Hz, those that were downregulated had high connectivity. *Camk2d* and *Mef2c* emerged as the most significantly downregulated (Figure 6 column 1; deep red).

The predominant response with low Hz stimulation compared to the control was downregulation of the hub genes *Igf1*, *Mef2c*, *Camk2d*, and *Gata4*. The *Nfatc* gene hub had high connectivity only for upregulation.

Taken together, the high frequency of *Mef2c* and *Igf1* targeting suggests that while these pathways are active modules, they are under intense miRNA-mediated “tuning” in the high and low Hz groups. Some of the downstream pathways affected by the hub gene *Mef2c* are wnt, notch, and calcium signaling families. Conversely, the key hub gene *Gata4* (55) was identified as a major node of inhibition release.

Finally, when comparing the two stimulation frequencies, high vs. low, it becomes apparent that the connectivity scores for downregulation and upregulation reflect similarly to those seen for high Hz vs. control. This may indicate that low Hz is more similar to the control. The hub gene *Edn1* (Endothelin-1, 56) was down in low Hz vs. control and upregulated in high Hz vs. low Hz (56). *Edn1* is a hub gene related to signaling through the adrenergic and calcium regulation families.

### 2.5. Treatment of EV-Naïve Myoblasts with Myotube EVs

**Figure 7:**
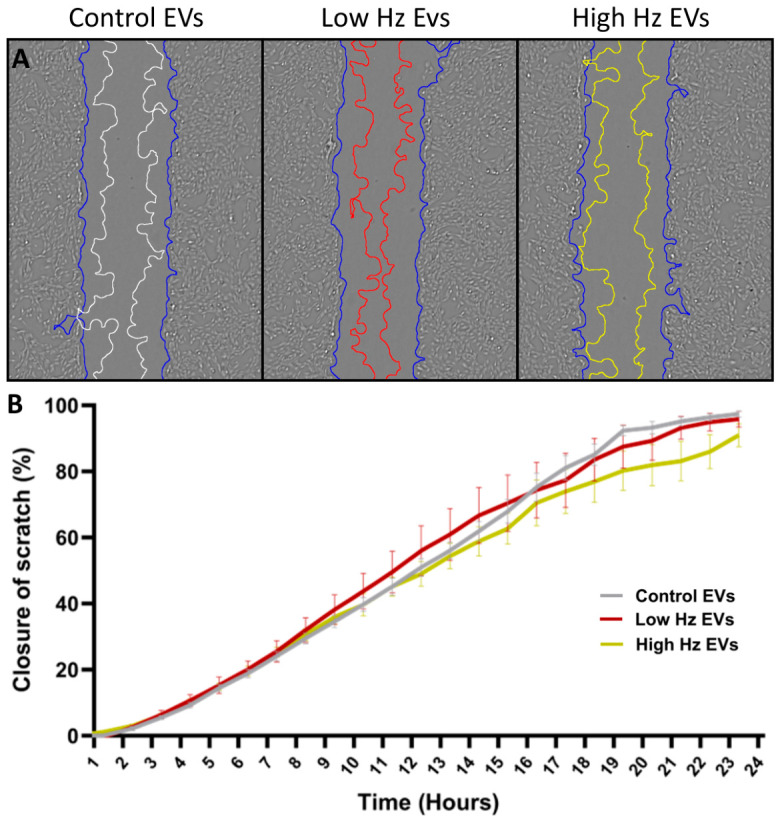
Functional assessment of effects of myotube EV treatment on myoblast migration. (**A**) Representative images of myoblast wound closure in response to small EV treatment at a concentration of 2.19 × 10^8^ (blue outline—0 H, color coded line—12 H post scratch). (**B**) The percentage of scratch closure over 24 h in response to EV treatment. Data expressed as the mean ± SEM. N = 5.

**Figure 8:**
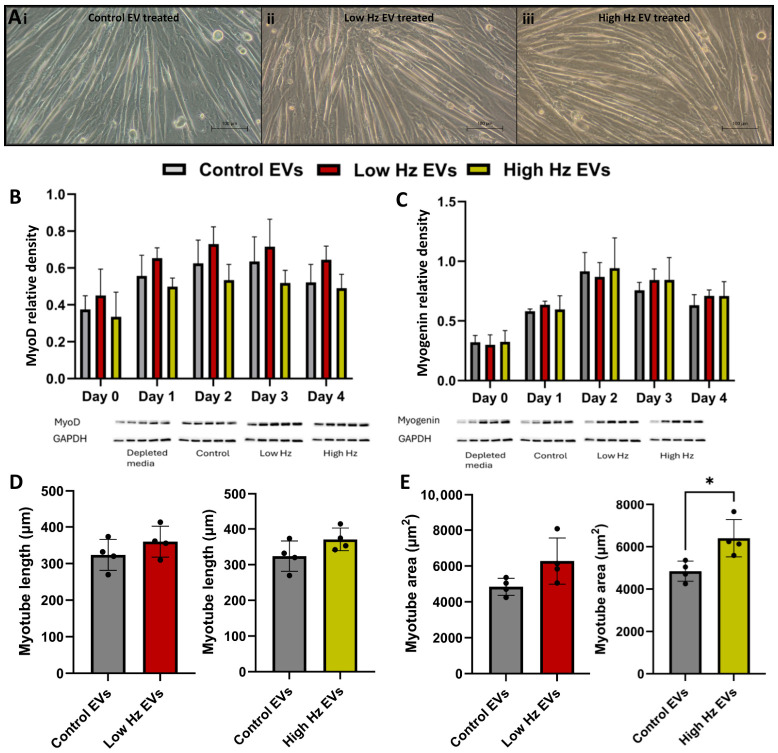
Functional assessment of effects of myotube EV treatment on myoblast differentiation. MyoD (**A**) and myogenin (**B**) levels in myotubes 4 days after culture in depleted media or treated with EVs (at a concentration of 2.19 × 10^8^ on day 0 and day 2). N = 2, no statistical analysis. (**C**) Representative images of myotubes after EV treatment. Scale bar—100 µm. (**D**) Average myotube length on day 4 of differentiation post-treatment. (**E**) Average myotube area on day 4 of differentiation post-treatment. Statistically significant differences were determined using a Student’s *t*-test. * = *p* < 0.05. N = 4.

Myotube-derived EVs from resting myotubes and from contracting myotubes were collected for characterization, an analysis of their miRNA cargo, and to be used as treatments. Four EV pellets from each EV group (control, low Hz stimulated, high Hz stimulated) were pooled and aliquoted to be used as EV treatments. C2C12 myoblasts were treated with these myotube-derived EVs at a concentration of 2.19 × 10^8^ EVs per mL, and the effect of these EVs on myoblast migration (Figure 7) and differentiation (Figure 8) was determined. Control EVs improved migration with significantly more scratch closure than depleted media at 15 (*p* < 0.05), 18 (*p* < 0.01), and 21 (*p* < 0.05) hours (see Supplementary file S2). Treatment with EVs harvested from myotubes exposed to EPS did not enhance migration compared to the addition of control EVs (see Figure 7B).

The expression of the myogenic regulatory factors MyoD and myogenin was evaluated via Western blotting over the course of differentiation (Figure 8B,C). These markers were selected as key regulators of myogenic commitment (MyoD) and differentiation (myogenin). Overall, MyoD expression followed a similar temporal pattern across all groups, while myogenin levels increased over the first two days of differentiation with a similar temporal pattern for all groups. These observations were limited to N = 2 experimental repeats and should be interpreted with caution.

Next, comparisons were made to determine if EVs from EPS-stimulated myotubes could alter myotube morphology overall within 4 days of treatment compared to control EVs. Supplementation with EVs from EPS-stimulated myotubes did not significantly affect myotube lengths, but high Hz stimulation increased the myotube area (*p* < 0.05) (Figure 8E). Together, these findings indicate that while myogenic regulatory factor expression remained largely similar between groups, EVs from the high Hz EPS group influenced myotube growth, suggesting an effect on later stages of myogenic maturation or fusion rather than early differentiation signaling.

## 3. Discussion

In this study, two different modes of electrical stimulation were applied to myotubes in culture. EVs were isolated from culture media and characterized, and their miRNA cargo was analyzed. EVs collected from the respective myotube groups were used as treatment to determine their effect on myoblast migration and differentiation. First, it was important to establish the optimal model, characterize the EVs using multiple methods, and determine the quantity and quality of the total RNA isolated. Second, the discussion will address the exploratory work on the miRNA cargo profiling and GO analysis of differentially expressed miRNAs.

### 3.1. EV Characterization

After isolation using differential ultracentrifugation, EVs were characterized according to the MISEV2018 guidelines [10]. There was no statistical difference in mean number and size between EVs isolated from resting versus EPS stimulated myotubes in response to the 10 h protocol (Figure 2C). This differs from the significant increase found by Murata et al. [33], who stimulated their myotubes for 24 h. During the 10 h post-EPS rest period, there appeared to be a change in the NTA size distribution graph (Figure 2B), where the EV levels in the low Hz stimulation group became more similar to the control. The number of EVs released by the high Hz group remained similar to the EV collection during the 10 h EPS protocol. A more comprehensive study titrating the EPS duration and subsequent non-stimulated resting time period could further delineate if these apparent trends can be confirmed. TEM micrographs show clearly defined negatively stained EVs of exosome size and morphology in the myotube EV pellets (Figure 2E). These look similar to those imaged by Pascucci and Scattini [42] using the same TEM preparation method. Media control pellets confirm EV depletion prior to culturing. Immunoblotting confirmed the presence of exosome markers Alix and CD81 in myotube EV pellets. Bands in the media control pellets mirror the NTA and TEM results. Depleted-media total RNA was much lower than the total RNA from myotube EVs (at least 9.58-fold lower in all three myotube EV groups). The depleted-media EV pellet also contained fewer miRNAs (96) compared to myotube groups’ EV pellets (324). This coincides with the EV depletion results and indicates that the majority of total RNA present in the myotube groups is packaged inside of the EVs. This highlights the importance of depleting EVs before culturing and performing sensitive miRNA sequencing.

### 3.2. MiRNA Sequencing

Among the top 50 most abundant miRNAs, several are known regulators of skeletal muscle differentiation, for example the myomiR **miR-206** [24]. Also playing a role in differentiation are **miR-1a**, a finding in experiments done both in vitro and in vivo [43], and **miR-26a**, as shown in developing mice and in adult mice with muscle injury and regeneration [44]. The same research group later demonstrated similar results for **miR-24** [45]. Also previously shown to influence differentiation in myoblasts are **miR-378b** [46] and **miR-133a** [47] and muscle development-associated **miR-615** [48], all of which were in the top 30. In slightly lesser amounts, but still in the top 50 and influencing differentiation, were **miR-22** [49] and **miR-183** [50].

Also present in the top 50 are miRNAs previously proven to play a role in satellite cell activation and entry into the myogenic program, as well as myoblast proliferation: **miR-99a/b** [51], **miR-320** [52], **miR-27a/b** [53,54], **miR-100** [55], all of which were in the top 30. The following were present in the top 50: **miR-125a** [56], **miR-148a** [57,58], and **miR-222** [59]. This could be interpreted as myotubes releasing EVs with miRs that signal other myoblasts in the vicinity or muscle cells elsewhere.

Taken together, of the miRNAs known to influence either differentiation or proliferation, **miR-206**, **miR-24**, **miR-148**, **miR-152**, and **miR-133a** were found to be increased by 25% or more in EVs from stimulated myotubes.

A few of the miRNAs present have been proven to be relevant for some clinical conditions, as shown in various experimental models. For example, it may be important that EPS increased miRNAs relevant to accelerated regeneration from injury and the amelioration of muscular dystrophy: **miR-378a/c/d** [60] and **miR-199a/b** [61]. A role in counteracting fibrosis has been demonstrated for **miR-21a** [62] and **miR-26** [63,64]. Although not in the top 30, two miRNAs in the top 50 are involved in GLUT4 signaling and glucose uptake in skeletal muscle, namely **miR-30d** [65,66] and **miR-182** [67].

Taking all of the above discussion together, three of the top five miRNAs present in the EVs released by the resting myotubes have been related to differentiation. These data provide further evidence for particular miRNA cargo that can explain the effect of myotube EV treatment on myoblasts that improves differentiation [16]. Also, amongst the prevalent miRNAs discussed above were several with the potential to have positive effects on clinical conditions (i.e., **miR-21**).

A noticeable finding was the number of miRNAs belonging to the let-7 family that were found in the top 10 (N = 5). Let-7 miRNAs were some of the earliest miRNAs to be discovered and were first discovered in *C. elegans* [68]. Later, let-7 miRNAs were found to be conserved across many species including humans [69]. Drummond et al. [70] found **let-7b** expression to be significantly higher in aged skeletal muscle compared to biopsies from younger participants and inversely correlated with pax7 expression, being low in aged muscle. They discovered that let-7-family members negatively regulate the cell cycle activators CDK6, CDC25A, and CDC34. This provides a possible mechanism for reduced satellite cell renewal in aged muscle. Conversely, all let-7 miRNA levels decreased in response to electrical stimulation indicating that EVs from exercised muscle could play a role in satellite cell activation. Let-7 negative fold differences in myotube-derived EVs were also found in a study by Murata et al. [33], who also electrically stimulated myotubes. Five of the let-7 miRNAs were downregulated in EVs from myotubes that underwent a 30 Hz EPS protocol. **Let-7e** was also discovered to regulate IGF2BP2, promoting muscle atrophy [71]. **Let-7e** expression was 0.61- and 0.59-fold lower in stimulation-group EVs compared to the control, indicating a role of contraction-derived EVs in myogenesis.

There was a large positive fold difference in both stimulation EV groups for several miRNAs not in the top 50 most present, and hence, the fold-change started from a lower baseline presence. **miR-23a** (positive FD of 2.06 in low Hz EVs and 3.31 in high Hz EVs) has been discovered to inhibit atrogin-1 and MURF1 expression in skeletal muscle, preventing muscle atrophy [72,73]. **miR-23b** (positive FD of 2.07 in low Hz EVs and 2.66 in high Hz EVs) is also linked to preventing muscle atrophy by upregulating MyHC expression, protein synthesis, and glucose uptake [74]. There is little to no literature exploring the roles of **miR-2137**, **miR-3473e/b**, and **miR-5128** in skeletal muscle development or function. They were upregulated in EVs from both stimulation groups and could provide new insight into muscle response to exercise and the role of muscle-released EVs in influencing other cell types.

In the literature, a few miRNAs have been shown to be differentially packaged into EVs after EPS in in vitro studies and after exercise in in vivo studies. Murata et al. [33] were the first to sequence myotube EV miRNA from control and EPS (30 Hz)-treated myotubes. They discovered that there were 10 miRNAs that had statistically significant positive fold differences and 7 that had negative fold differences in EPS EVs. When assessing the similarities between that study and the current study’s results, positive fold differences in common with the current study include **miR-206**, and negative fold differences in common include five members of the let-7 family (**let-7f/a/d/e/g**). Lautaoja-Kivipelto et al. [75] also sequenced the miRNA from C2C12 myotube EVs with **miR-206** being the most present miRNA. They discovered that after low Hz EPS, there was a positive fold difference in stimulated EVs compared to the control for **miR-206** (*p* = 0.074) and **miR-1** (*p* < 0.05). **miR-133a** displayed a numeral increase in stimulated EVs, though it was not statistically significant due to inter-sample variation. These myomiR increases were also found in the current study. These two in vitro studies did not use depleted media, which means that some of the miRNA sequenced could be from FBS EVs. For example, **miR-423** is reported to be present in myotube EVs in the study by Murata et al. [33]; this miRNA was not highly present in the myotube EVs of the current study, though it is the second most present in the depleted media EV pellet. This could indicate that not depleting media of EVs could be a source of miRNA cross contamination.

In vivo studies in humans have been used to study EV-packaged miRNA changes in response to exercise. Annibalini et al. [76] discovered an increase in plasma EV-associated **miR-206** and **miR-146a** levels after two hours of intermittent resistance exercise. This is reflected in the current study with **miR-206** (ranked 1st) and **miR-146b** (ranked 71st) having a slight positive fold difference in EVs from contracting myotubes. Likewise, Guescini et al. [77] discovered that 40 min of high-intensity aerobic exercise increased **miR-133b** and **miR-181a** levels within circulating EVs. In the current study, **miR-133b** (ranked 88th) had a large positive fold difference in both stimulation groups compared to control EVs. These comparisons provide further rationale for using in vitro exercise models for validating aspects of the muscle-specific secretome, including a role for EVs in adaptation to exercise.

**miR-1** has been proven to be downregulated in response to mechanical overloading in rodent models [21]. Along with evidence that **miR-1** targets pro-growth genes including IGF-1 and Cyclin D [78,79], this indicates a major role in muscle hypertrophy regulation. Fei et al. [80] further proved this point by revealing that preventing **miR-1** downregulation stunts hypertrophy in rodents. **miR-1-a** (ranked 3rd) displayed negative fold differences in both of the stimulated myotube EV groups compared to the control (low Hz FD: 0.64; high Hz FD: 0.74). These differences reflect the downregulation of **miR-1** in skeletal muscle after exercise providing confirmation of the rodent studies.

Mesenchymal stem cells (MSCs) have long been used in stem cell therapy, and more recently, MSC-derived EVs have been shown to ameliorate muscle damage in ischemic mice models, including hindlimb ischemia [81,82]. Nakamura et al. [83] sequenced the miRNA from MSC EVs and found **miR-125b-5p**, **miR-25-3p**, and **miR-23a-3p** in the top 20 most present miRNAs. These miRNAs were found to be present in the myotube EVs of the current study (**miR-125b**—47th, **miR-25-3p**—56th, **miR-23a-3p**—64th). Since the levels were not amongst the more prevalent, this confirms the cell-specificity of EV miRNA cargo. However, they have been discovered to relieve skeletal muscle atrophy (**miR-125b** [84], **miR-23a** [73]).

### 3.3. Gene Ontology

The high-resolution mapping of miRNA–mRNA networks demonstrated that the miRNA response to electrical stimulation is likely not a stochastic byproduct of cellular stress, but rather a coordinated regulatory program targeting critical signaling hubs [85]. By releasing EVs containing cargo that included this regulatory interface, C2C12 myotubes could signal other cells in the culture.

This study revealed some intensity-dependent shifts in key myogenic miRNA clusters. The identification of these muscle-specific miRNA shifts aligns with established paradigms where miRNAs govern the critical transition between myoblast proliferation and terminal differentiation [86]. Notably, the GO analysis revealed that overarching processes such as “muscle cell differentiation” were targeted for both activation and suppression simultaneously within the high Hz group. Rather than a contradiction, this may reflect orchestrated muscle plasticity; EVs act as rheostats to fine-tune the timeline of regeneration, simultaneously dampening earlier proliferative pathways while derepressing late-stage structural assembly [87].

In the low Hz vs. control comparison, the network mapping highlighted a primary focus on targeting of the *Igf1* and *nfatc3* genes and associated pathways and networks, which may be interpreted as maintaining baseline anabolic potential whilst improving calcium handling and metabolic efficiency via the sarcoplasmic reticulum SERCA pump. [88]. By prioritizing these pathways, the low Hz EVs favor ATP efficiency in recipient cells.

Conversely, high Hz stimulation resulted in more shifts in the pathways connected to the identified gene hubs. It would appear that the regulatory focus shifted toward structural proteostasis, morphogenesis, and satellite cell lineage progression [89]. The signature revealed in the heatmap suggests a targeted release of inhibition on central growth pathways that was less evident with low Hz stimulation. This could be interpreted as the secreting myotubes amplifying the recipient cells’ capacity for protein synthesis and hypertrophy in response to anabolic growth factors.

A central finding across the comparative heatmap was the role of *Mef2c* as a master regulatory hub. *Mef2c* is a critical transcription factor controlling sarcomeric assembly, metabolic gene expression, and fiber-type switching [90]. In the high Hz vs. control suppression profile, its connectivity to dozens of targeted pathways suggests that EV-miRNAs released following EPS were deployed to fine-tune *Mef2c*-driven transcription. We hypothesize that this regulatory focus served as a compensatory “braking system” to prevent unchecked, aberrant muscle growth or premature fiber-type switching under high-intensity stimulation.

The direct comparison of high Hz vs. low Hz conditions provided evidence for an intensity-associated influence on miR hubs. While the high Hz EVs promoted signals for mass accumulation (morphogenesis), they simultaneously suppressed calcium and adrenergic signaling families relative to the low Hz group. Calcium signaling is integral to the efficiency of the contractile apparatus [91]. This divergence may indicate that a high-intensity stimulus can successfully promote hypertrophic mass, but it does so at the temporary expense of contractile efficiency, as evidenced by the targeting of calcium-handling hubs such as *Camk2d* [92]. In conclusion, given the GO results, we propose that EV-packaged miRNA alters the downstream effect of the muscle EV secretome, routing signaling through specific developmental and structural cascades under high demand. The miRNA secretomes resulting from different stimulation regimens appeared to have distinct influences on the myotube area. These findings are associative, and further temporal and functional validation would be required to more definitively establish specific miR–mRNA target interactions.

The identification of hubs such as *Mef2c* (Connectivity Score: 62–65) demonstrates the possibility that the EV cargo is not merely altering a list of genes, but is systematically targeting high-traffic nodes in the myogenic signaling network. This topological connectivity provides a preliminary map of how EPS pacing may coordinate physiological remodeling pathways.

A limitation of this study is that EV samples used for miRNA sequencing were pooled across biological replicates prior to sequencing. While this approach enabled sufficient RNA input and provided a robust overview of EV-associated miRNA cargo within each experimental condition, it precludes the statistical testing of differential miRNA abundance at the individual miRNA level. Therefore, sequencing data should be interpreted as a representative and compositional profile of EV miRNA cargo rather than as a replicate-based differential expression analysis. Future studies incorporating replicate-level small RNA sequencing will be required to validate condition-specific changes in EV miRNA abundance.

### 3.4. EV Treatments

EV treatments of 2.19 × 10^8^ EVs per mL had positive effects on myoblast migration and differentiation when compared to the EV-depleted condition (Figure 7 and Figure 8). Further, EV treatment with EVs harvested from the high Hz group increased the myotube area (vs control EVs). The trend was less convincing for low Hz treatment (*p* value = 0.0816). However, these results form a basis for further experimentation. Aswad et al. [93] demonstrated that depleted media inhibit proliferation, which is in line with results from the current study. The positive effect of myotube EVs on differentiation has also been demonstrated by Forterre et al. [16]. The current study utilized a concentration of 2.19 × 10^8^ EVs/mL, which is lower than the 1 × 10^9^ to 1 × 10^10^ EVs/mL that has previously been shown to improve the differentiation of myoblasts when the EVs were harvested from resting myotubes [16]. Treatment at a higher EV concentration may lead to greater differences in treated myoblasts between contraction-derived EVs and control EVs. Guescini et al. [94] treated myoblasts with EVs from control and oxidatively injured myotubes, with EVs from injured myotubes resulting in increased myoblast proliferation and decreased differentiation. The treatment concentration of 1 × 10^10^ EVs per mL was 45 times greater than the one used in the current study, which harvested EVs for multiple purposes. Chang et al. [95] likewise found EV treatments to be increasingly effective at ameliorating muscle atrophy at higher concentrations with the highest concentration being 1 × 10^9^ EVs per mL.

These findings suggest that while the molecular “message” (miRNA cargo) was remodeled by EPS, the concentration used in our in vitro validation may have been too low to fully elucidate potential phenotypic manifestations, highlighting the need for future dose–response titrations.

## 4. Materials and Methods

### 4.1. Cell Culture

Before seeding, 6-well culture dishes (NEST Biotechnology; Cat. No: 703001) (Wuxi, China) were coated with Geltrex (Gibco; Cat. No: A14132-02) (Grand Island, NY, USA) (diluted *v*/*v* 1:100 in DMEM) for 1 h at 37 °C. Murine C2C12 myoblasts (ATCC^®^, Cat. No: CRL-1772TM) (Manassas, VA, USA) were then seeded into 6-well culture dishes at 15,000 cells per cm^2^ in normal growth media (DMEM (Gibco; Cat. No: 41965039); 2% *v*/*v* Pen strep (Gibco; Cat. No: 15140122); 2% *v*/*v* Glutamax (Gibco; Cat. No: 35050061); 10% *v*/*v* fetal bovine serum (FBS) (Gibco; Cat. No: 10493106), and cells were cultured at 37 °C with 5% CO_2_. At 90% confluency, the media were changed to differentiation media and the cells were left to differentiate for 5 days (DMEM; 2% *v*/*v* Pen strep; 2% *v*/*v* Glutamax; 2% *v*/*v* Horse serum (Celtic Molecular Diagnostics; Cat. No: S0910) (Cape Town, South Africa)). The media volumes were 2 mL per well for proliferation and differentiation growth phases.

### 4.2. Depleting FBS of Extracellular Vesicles

Serum is an abundant source of EVs and so before using fetal bovine serum (FBS) in culture media for an EV collection experiment, the EVs need to be removed. To achieve this, FBS was centrifuged at 100,000× *g* for 16 h at 4 °C (Optiseal tubes, Beckman Coulter; Cat. No: 361625) (Brea, CA, USA) in an ultracentrifuge (Optima Ultracentrifuge, Beckman Coulter; type 70ti fixed-angle rotor). Centrifuging at this speed and duration has been shown by Théry et al. [96] to be optimal for EV removal. After centrifuging, the top 20 mL of supernatant was removed and filtered through a 0.22 µm PVDF syringe-driven filter (Millex, Millipore; Cat. No: SLGV033RS) (Burlington, MA, USA).

### 4.3. Electrical Pulse Stimulation

On day 5 of differentiation, 2.5 mL of EV-depleted media were added to each well and the EPS was applied to the myotubes using the C-Pace electrical pulse stimulator (IonOptix, Dublin, Ireland). There were two stimulation groups along with a control group receiving no stimulation. The low Hz cells received constant electrical stimulation with pulses of 2 ms, 13 V, and 2 Hz. The high Hz group received pulses of 2 ms and 13 V with a frequency of 30 Hz for 5 s, 5 s rest, 5 Hz for 5 s, and then 5 s rest (adapted from Orfanos et al. [32]). There were two culturing periods: 10 h of EPS, media change and then 10 h of rest. View videos of myotubes being stimulated with EPS by accessing Supplementary file S3.

### 4.4. Extracellular Vesicle Isolation from Conditioned Media

Conditioned media were subjected to differential ultracentrifugation to isolate the EVs released from the myotubes. The first two centrifuge steps are 300× *g* for 10 min and 2000× *g* for 20 min to remove large cellular debris. The last two steps make use of an ultracentrifuge (Beckman Coulter) and a type 70ti rotor (Beckman Coulter), they are 10,000× *g* for 30 min and 100,000× *g* for 2 h at 4 °C and make use of Optiseal polypropylene centrifuge tubes (Beckman Coulter, Cat. No: 361625). The small EV pellet that forms after the last round of centrifuging was resuspended in 400 µL of PBS and stored at −80 °C in Protein LoBind 1.5 mL tubes (Eppendorf, Cat. No: 0030108116) (Hamburg, Germany).

### 4.5. Nanoparticle Tracking Analysis

Nanoparticle tracking analysis (NTA) was done on individual EV pellets before pooling for statistical analysis purposes, and further NTA was performed on pooled EV pellets to get the total EV count to calculate treatment concentrations (Nanosight LM10, Malvern Panalytical, Malvern, UK). Five videos of 60 s each were taken for each sample for analysis using NTA software (Version 3.2). For video acquisition, the camera level was set to 16 and the screen gain was set to 10; for video analysis, the detection threshold was set to 6. The concentration was reported in particles per mL and size was reported in nanometers.

### 4.6. Western Blotting

#### 4.6.1. Extracellular Vesicle Blotting

Western blotting was performed on myotube-derived extracellular vesicle pellets, as well as control media pellets. EV pellets were prepared for Western blotting by adding 5× RIPA (50 mM Tris-HCL, 150 mM NaCl, 1% Triton X-100, 0.25% Sodium deoxycholate, 1 mM EDTA, pH 7.4) to a final concentration of 1× along with a protease inhibitor (Roche; Cat. No: 04906837001) (Basel, Switzerland). For myotube EV groups, samples were loaded by particle number (4.8 × 10^9^ EVs per well). For media controls, samples were loaded by equal volumes (120 µL per well). Samples were denatured with 4× Laemmli buffer (62.5 mM Tris-HCL, pH 6.4, 2% (*w*/*v*) SDS, 10% (*v*/*v*) glycerol, 0.005% (*w*/*v*) bromophenol blue, 10% (*v*/*v*) β-mercaptoethanol) and boiled for 5 min at 95 °C. A PageRuler prestained protein ladder was used to determine protein sizes (Thermo Fisher; Cat. No: 26620) (Waltham, MA, USA). Blotting membranes were blocked with 10 mL of EveryBlot blocking buffer for 10 min (BioRad; Cat. No: 12010020) (Hercules, CA, USA). Primary antibodies of pan-EV markers (diluted 1:500 (*v*/*v*) in TBS-T with 1% (*w*/*v*) BSA) were incubated overnight at 4 °C, antibodies used were Alix—(Santa Cruz; Cat. No: SC53538) (Dallas, TX, USA) and CD81 (Santa Cruz; Cat. No: SCBSC13118). A further 4 h incubation in primary antibodies at room temperature followed the next day. After washing, the membranes were incubated in an HRP-linked secondary antibody for 1 h at room temperature. Membranes were then washed 7 × 5 min in TBS-T. After incubating the membranes in a maximum sensitivity ECL reagent (SuperSignal West Femto Maximum Sensitivity Substrate, Thermo Fisher; Cat. No: 34096), the ChemiDoc Imaging System (BioRad, USA) was used to visualize chemiluminescent bands.

#### 4.6.2. Myotube Lysate Blotting

This was a quantitative analysis to determine the effect of EV treatments on myoblast differentiation over 4 days. RIPA buffer was added to cells, and a cell scraper (NEST; Cat. No: 710001) was used to remove myotubes from the culture plates. Cell lysates were centrifuged at 16,000× *g* for 10 min, and the supernatant collected. The Micro BCA protein assay (Thermo Fisher; Cat. No: 23235) was used to determine protein concentrations of samples; 15 µg of protein was loaded into each well (10-well comb, Bio-Rad; Cat. No: 1653365). The same steps were followed as above in the EV blotting procedure. Primary antibodies used were as follows: Myogenin (Invitrogen; Cat. No: 14 5643-82) (Carlsbad, CA, USA) (1:500 (*v*/*v*) in TBS-T with 1% BSA), MyoD (Dako; Cat. No: M3512) (Glostrup, Denmark) (1:500 (*v*/*v*) in TBS-T with 1% BSA). After chemiluminescent imaging, membranes were stripped with a mild stripping buffer and re-probed for GAPDH, which was used as an internal loading control for the cell lysate blots.

### 4.7. Electron Microscopy

#### 4.7.1. Transmission Electron Microscopy

Carbon-coated copper grids (Agar Scientific, Sheffield, UK) were rendered hydrophilic using a EMS100 Glow Discharge Unit (Electron Microscopy Sciences, Morgantown, PA, USA). Droplets of each of the myotube-derived EV samples, as well as from control media samples, were placed on the grids and negatively stained with 2% uranyl acetate (SPI Supplies, West Chester, PA, USA). Transmission electron microscopy imaging was done using a FEI Tecnai F20 transmission electron microscope (Thermo Fisher (FEI), Eindhoven, The Netherlands) operating at 200 kV. Images were collected using a DE-16 camera (Direct Electron, San Diego, CA, USA).

#### 4.7.2. Scanning Electron Microscopy

Myotubes on day 5 of differentiation were resin-embedded (SEM preparation protocol adapted from Graham and Orenstein [97]), and ultrathin sections of 100 µm were cut with a Leica UC7 ultramicrotome system (Leica Microsystems, Wetzlar, Germany) and a 45° Diamond knife (Diatome) and picked up on 300 mesh nickel grids. Sections were viewed using the Apreo Volumescope scanning electron microscope (Thermo Fisher, Eindhoven, The Netherlands), and images were acquired using the Xt Microscopy (Thermo Fisher) software (Version 13.9.1).

### 4.8. Extracellular Vesicle Micro-RNA Cargo Analysis

Pooled samples (with a volume of 200 µL—representative of 12 × 6-well plates worth of conditioned media) of each of the myotube-derived EV groups (control, low Hz, and high Hz) were sent to Macrogen Inc. (Seoul, South Korea) for EV miRNA cargo analysis; a depleted media control sample was sent for analysis as well. The RNA concentration was determined using the Qubit 4 model (Thermo Fisher). MiRNA was extracted using the miRNeasy Serum/plasma advanced kit (QIAGEN; Cat. No: 217084) (Venlo, The Netherlands). A Bioanalyzer RNA Pico 6000 chip was used to determine RNA quality. Library preparation was performed as follows: concentration measured via qPCR, size check using a Bioanalyzer DNA High Sensitivity chip kit, NEBNext^®^ Multiplex Small RNA Library Prep Set for Illumina^®^ (New England Biolabs; Cat. No: E7560S) (Ipswich, MA, USA). The NEBNext Multiplex Small RNA Library Prep Set for Illumina contains the adaptors, primers, enzymes, and buffers required to convert small RNAs into indexed libraries for next-generation sequencing on the Illumina platform.

### 4.9. Gene Ontology Analysis

#### 4.9.1. miRNA Quantification and Normalization

Bioinformatic analysis was performed using RStudio (Build 418). RNA sequencing was performed on samples pooled from 12 biological replicates per experimental group.

Total miRNA expression was analyzed using the edgeR Bioconductor package [98]. Normalization was conducted using the trimmed mean of M-values (TMM) method. To ensure high-quality data, a low-expression filter was implemented, retaining only miRNAs with a threshold of >15 counts per million in at least one experimental group. All downstream calculations were performed on log_2_-transformed normalized counts.

#### 4.9.2. Differential Expression (DE) Criteria

TMM-normalized counts were then utilized to calculate differential expression across six comparative groups: high Hz vs. control (UP/DOWN), low Hz vs. control (UP/DOWN), and high Hz vs. low Hz (UP/DOWN). MiRNAs were categorized based on their expression relative to the control or a comparison group using specific log_2_ fold-change values. Upregulated miRNAs were defined by a stringent log_2_FC > 1 (>2-fold change), while downregulated miRNAs were defined by a log_2_FC < −1.

#### 4.9.3. Integrative miRNA–mRNA Target Prediction

High-confidence mRNA targets for *Mus musculus* were identified using the multiMiR package [99]. Targets were aggregated from three validated databases: miRTarBase (experimentally validated), TarBase (experimentally supported), and miRDB (high confidence predicted). mRNA symbols were cross-referenced with the org.Mm.eg.db database to ensure nomenclature accuracy.

#### 4.9.4. Functional Enrichment and Hub Mapping

Gene Ontology Biological Process enrichment was conducted via clusterProfiler [100]. An initial, relaxed *p*-value cutoff of 0.5 was intentionally utilized strictly as an intermediate data-extraction step to capture broad signaling network topologies. This exploratory parameter prevented the premature exclusion of critical intermediate signaling nodes (false negatives) required for comprehensive network mapping. Results were filtered for muscle-specific keywords including “satellite cell,” “myoblast,” “myogenic,” “sarcomere,” “actin filament,” “muscle fusion,” and “skeletal muscle”. Gene ratios for these pathways were calculated as the number of genes of interest in the pathway divided by the total number of genes in that pathway. To interpret the high-volume output of the Gene Ontology (GO) enrichment and identify complex molecular patterns, the large language model Gemini 3.1 Pro () was utilized as an analytical tool. This workflow facilitated the identification of master regulatory hub genes by ranking mRNA targets according to the total number of discrete biological processes they influence. In this network approach, stochastic false positives fail to cluster, whereas true biological signals converge into highly connected master regulatory hubs.

These hubs were subsequently mapped to their overarching signaling families (e.g., Wnt, MAPK, IGF-1). The analytical pipeline consisted of three primary steps: (1) semantic clustering of related GO terms into broader functional signaling families; (2) the application of directionality logic to classify pathways as either “derepressed” (activated) or “silenced” (suppressed) correlating with the differential expression of targeting miRNAs; and (3) cross-comparison of experimental conditions to distinguish between shared regulatory hubs and intervention-specific gene networks. View details of GO analysis and hub mapping by accessing Supplementary file S4.

#### 4.9.5. Statistical Visualization

Data visualization was performed using ggplot2, patchwork, and scales. To address multiple hypothesis testing and control the false discovery rate for the broad GO enrichment output, visualization plotting was capped at a statistical threshold. All final targeted GO pathways presented as significant utilized the Benjamini–Hochberg False Discovery Rate (*q* < 0.05). Enrichment dot plots display the gene ratio on the x-axis against biological processes, with significance color-scaled by the adjusted *p*-value. For target frequency plots, the top mRNA targets (head 20) were visualized via bar plots, with the y-axis representing the target frequency.

#### 4.9.6. Functional RStudio Script

An Rstudio script for the miRNA analysis workflow is available in Supplementary file S5. Minor adjustments will need to be made regarding the names of the raw data files if this script is to be reused, i.e., the names of the files being analyzed must match those seen in the script. The raw data files will need to be Excel files with a specific layout to be worked on by the script. An example of the Excel sheet layout is also included (Supplementary file S6).

### 4.10. Functional Assessment of EVs—EV Treatments

#### 4.10.1. Migration Assay

C2C12 myoblasts (passage 10) were seeded at 10,000 cells per cm^2^ in 24-well culture dishes (SPL Life Sciences; Cat. No: 30024) (Gyeonggi-do, Republic of Korea) in normal growth media and were cultured at 37 °C and 5% CO_2_ for 24 h. Once confluent, cells were scratched using an SPL scar scratcher (SPL Life Sciences; Cat. No: 201925) to create a relatively uniform scratch. The cells were washed with DMEM to remove debris, and then, 400 µL of depleted media containing 1 µg/mL Mitomycin C (Roche; Cat. No: 10107409001) was added to prevent proliferation. Myoblasts were treated with C2C12 myotube-derived EVs from the respective groups at a concentration of 2.19 × 10^8^ EVs per mL. The 24-well plate was placed onto an in-incubator imaging system (Olympus Provi CM20, Olympus, Tokyo, Japan). Images of the scratch area were taken every 20 min for 24 h. Images were analyzed using ImageJ (Version 1.54p) with the use of the wound healing plugin. The scale was set to 0.46 pixels/µm. The settings used with the wound healing plugin were as follows: variance set to 20, threshold value set to 50, and the percentage saturated pixels set to 0.001%.

#### 4.10.2. Differentiation Assay

C2C12 myoblasts (passage 10) were seeded at 15,000 cells per cm^2^ in 12-well culture plates (NEST; Cat. No: 712001) in normal growth media and cultured at 37 °C and 5% CO_2_. After 24 h, cells were washed and switched to depleted media and treated with C2C12 myotube-derived EVs from each group at a concentration of 2.19 × 10^8^ EVs per mL. One well was not treated with EVs and served as a control. Cell media were refreshed after 2 days, and an identical second EV treatment was added to the cells. Phase contrast images were taken each day, and cell lysates were prepared for Western blotting to determine the effects of EV treatment on differentiation.

### 4.11. Use of Large Language Models (LLMs)

During the preparation of this manuscript, the authors utilized large language models (LLMs) exclusively to improve grammatical accuracy, enhance readability, and condense lengthy sections of text. These AI-assisted technologies were employed strictly as language-editing tools and were not used to generate novel scientific insights or formulate conclusions. Following the use of these tools, the authors thoroughly reviewed, edited, and verified all text.

### 4.12. Statistical Analysis

Statistical analyses included a one-way and two-way ANOVA with multiple comparisons and were performed with GraphPad prism (Version 10.0.0). Data was found to be normally distributed. All data are represented as means ± standard errors of the mean. *p*-values of less than 0.05 were considered statistically significant.

## 5. Conclusions

This study provides a comprehensive view of the effect of EPS on the miRNA packaged into myotube EVs. Many muscle-important miRNAs were packaged into myotube EVs, as well as many miRNAs whose function have only recently been discovered. Both low Hz and high Hz stimulation proved to alter miRNA levels in the EVs released into culture media. GO analysis based on differentially expressed miRNAs in the EVs of the EPS-exposed groups indicates distinct EV signaling in response to low- and high-frequency stimulation. This could provide evidence as to how SkM EV signaling contributes to adaptations to different exercise modalities. Findings from this study highlight the importance of in vitro models in studying the SkM-specific EV response to contraction. Using this platform of miR data, future studies could investigate miRNAs that emerged as potentially interesting. Such studies could include in vitro verification or miR analysis of circulating EVs in blood samples from human participants with exercise intervention. A comparison of miR cargo from all isolated EVs should be compared to miR cargo of EVs sorted for a muscle marker. This remains an urgent technical need in the field.

## Figures and Tables

**Table 1:** Top 30 miRNAs present in myotube EVs with fold changes of stimulation groups compared to unstimulated control.

Rank	miRNA	Control TMM	Low Hz TMM	High Hz TMM	Low Hz Fold Change	High Hz Fold Change
1	miR-206-3p	342,377	397,949	478,669	1.16	1.40
2	miR-378a-3p	71,599	80,637	86,447	1.13	1.21
3	miR-1a-3p	56,905	36,453	41,928	0.64	0.74
4	let-7c-5p	40,238	37,980	34,416	0.94	0.85
5	miR-21a-5p	31,768	29,417	27,469	0.93	0.87
6	let-7f-5p	31,190	25,783	25,425	0.83	0.82
7	miR-378d	22,959	26,441	24,844	1.15	1.08
8	let-7a-5p	16,117	12,524	11,483	0.78	0.72
9	let-7b-5p	12,007	11,423	9488	0.95	0.79
10	let-7i-5p	10,359	11,983	8541	1.16	0.83
11	miR-378c	10,216	11,490	11,533	1.13	1.13
12	miR-26a-5p	9825	9084	8425	0.93	0.86
13	miR-486a-5p	18,428	8002	8326	0.43	0.45
14	miR-486b-5p	18,782	7780	7910	0.41	0.42
15	miR-128-3p	6499	6320	7012	0.97	1.08
16	miR-99a-5p	6839	6513	6647	0.95	0.97
17	miR-320-3p	6323	4783	6680	0.76	1.06
18	miR-143-3p	5294	4638	3689	0.88	0.70
19	miR-99b-5p	5231	3024	3406	0.58	0.65
20	miR-532-5p	4598	5431	5716	1.18	1.24
21	let-7d-5p	4963	2986	3090	0.60	0.62
22	miR-196a-5p	3870	2744	2609	0.72	0.64
23	miR-27b-3p	3768	3595	3290	0.95	0.87
24	let-7g-5p	3461	2957	2393	0.85	0.69
25	miR-24-3p	3483	3962	5168	1.14	1.48
26	miR-30d-5p	3346	2937	2891	0.88	0.86
27	miR-378b	3332	3073	3340	0.92	1.00
28	miR-615-3p	2998	1179	1362	0.39	0.46
29	miR-501-3p	2668	2068	3223	0.78	1.21
30	miR-27a-3p	2743	2396	1911	0.87	0.70

Footnote: TMM = trimmed mean of M-values for single sample representative of EVs from 12 × 6-well plates pooled. Fold changes N = 1 per group. Green—positive change compared to control. Red—negative fold change compared to control.

**Table 2:** MyomiR cargo of myotube EV groups.

Rank	miRNA	Control TMM	Low Hz TMM	High Hz TMM	Low Hz Fold Change	High Hz Fold Change	Known Skeletal Muscle Function
1	miR-206-3p	342,377	397,949	478,669	1.16	1.40	Differentiation
3	miR-1a-3p	56,905	36,453	41,928	0.64	0.74	Differentiation
13	miR-486a-5p	18,428	8002	8326	0.43	0.45	Migration, Fusion, Differentiation
14	miR-486b-5p	18,782	7780	7910	0.41	0.42	Migration, Fusion, Differentiation
50	miR-133a-3p	1045	2967	2243	2.84	2.15	Proliferation, Fusion, Differentiation
88	miR-133b-3p	234	1662	931	7.08	3.97	Fusion, Differentiation

Footnote: Green—positive change compared to control. Red—negative fold change compared to control.

## Data Availability

The data that support the findings of this study are available from the corresponding author upon request.

## Supplementary Materials

The following supporting information can be downloaded at: https://www.mdpi.com/article/10.3390/ijms27104320/s1.

## Abbreviations

The following abbreviations are used in this manuscript:

AMPK	AMP-activated protein kinase
ECL	Entactin-collagen IV-laminin
EPS	Electrical pulse stimulation
EV	Extracellular vesicle
GLUT4	Glucose transporter 4
IL-6	Interleukin-6
miRNA	Micro-RNA
MSC	Mesenchymal stem cell
mTOR	Mammalian target of rapamycin
MVB	Multivesicular body
NTA	Nanoparticle tracking analysis
piRNA	PIWI-interacting RNA
SkM	Skeletal Muscle
snoRNA	Small nucleolar RNA
snRNA	Small nuclear RNA
TMM	Trimmed mean of M-values
