# Supplementary material for: Small Extracellular Vesicle Release Following Electrical Pulse Stimulation of C2C12 Myotubes: Effects on microRNA Cargo and Myoblast Migration and Differentiation
Source: Int J Mol Sci. 2026 May 12;27(10):4320. doi: 10.3390/ijms27104320 (PMC13206931; doi:10.3390/ijms27104320)
Supplement: Supplementary file 1 [file ijms-27-04320-s001.zip › ijms-4231158-supplementary/Supplementary file 1 - EV miRNA analysis/Sequencing report - Mus musculus - Myotube EV groups.pdf]

Research use only

Mus musculus
miRNA Sequencing

Report

November 2024

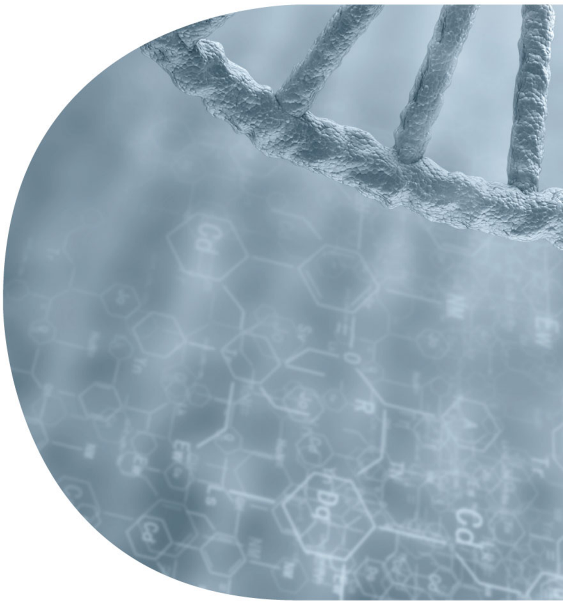

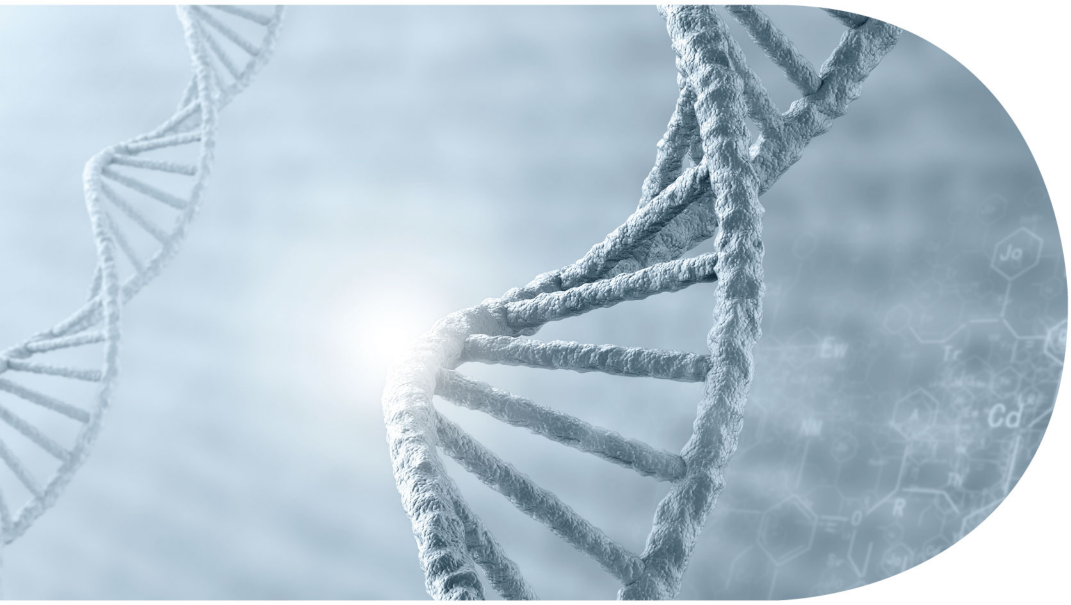

ver. MGSR3.0_mm10

Copyright © Macrogen

Project Information

Client Name	Macrogen Europe
Company/Institution	Macrogen Europe
Order Number	HN00227484
Species	*Mus musculus*
Reference	mm10
miRBase / RNAcentral version	miRBase v22.1 / RNAcentral 14.0
Type of Read	Paired-ends
Read Length	101
Number of Samples	3
Library Kit	NEBNext Small RNA Library Prep Set for Illumina Kit
Library Protocol	Protocol for use with NEBNext Small RNA Library Prep Set for Illumina
Type of Sequencer	Illumina platform

Table of Contents

Project Information 02

1. Experimental Methods and Workflow 04

2. Analysis Methods and Workflow 06

3. Summary of Data Production 08
- 3. 1. Raw Data Statistics 08
- 3. 2. Average Base Quality at Each Cycle 09
- 3. 3. Trimming Data Statistics 10
- 3. 4. Average Base Quality at Each Cycle after Trimming 13
- 3. 5. Final processed reads after removing unwanted reads 14
- 3. 6. Read length distribution of each sample 15

4. miRNA Analysis Result 16
- 4. 1. Summary of Small RNA Composition 16
- 4. 2. Quantification of Mature miRNA Abundance 17
- 4. 3. Prediction of known/novel miRNA 22
- 4. 4. Small RNA Composition 33

5. Data Download Information 35
- 5. 1. Raw Data 35
- 5. 2. Analysis Results 35

6. Appendix 38
- 6. 1. Phred Quality Score Chart 38
- 6. 2. Programs used in Analysis 39
- 6. 3. References 41

Humanizing Genomics macrogen

1. Experimental Methods and Workflow

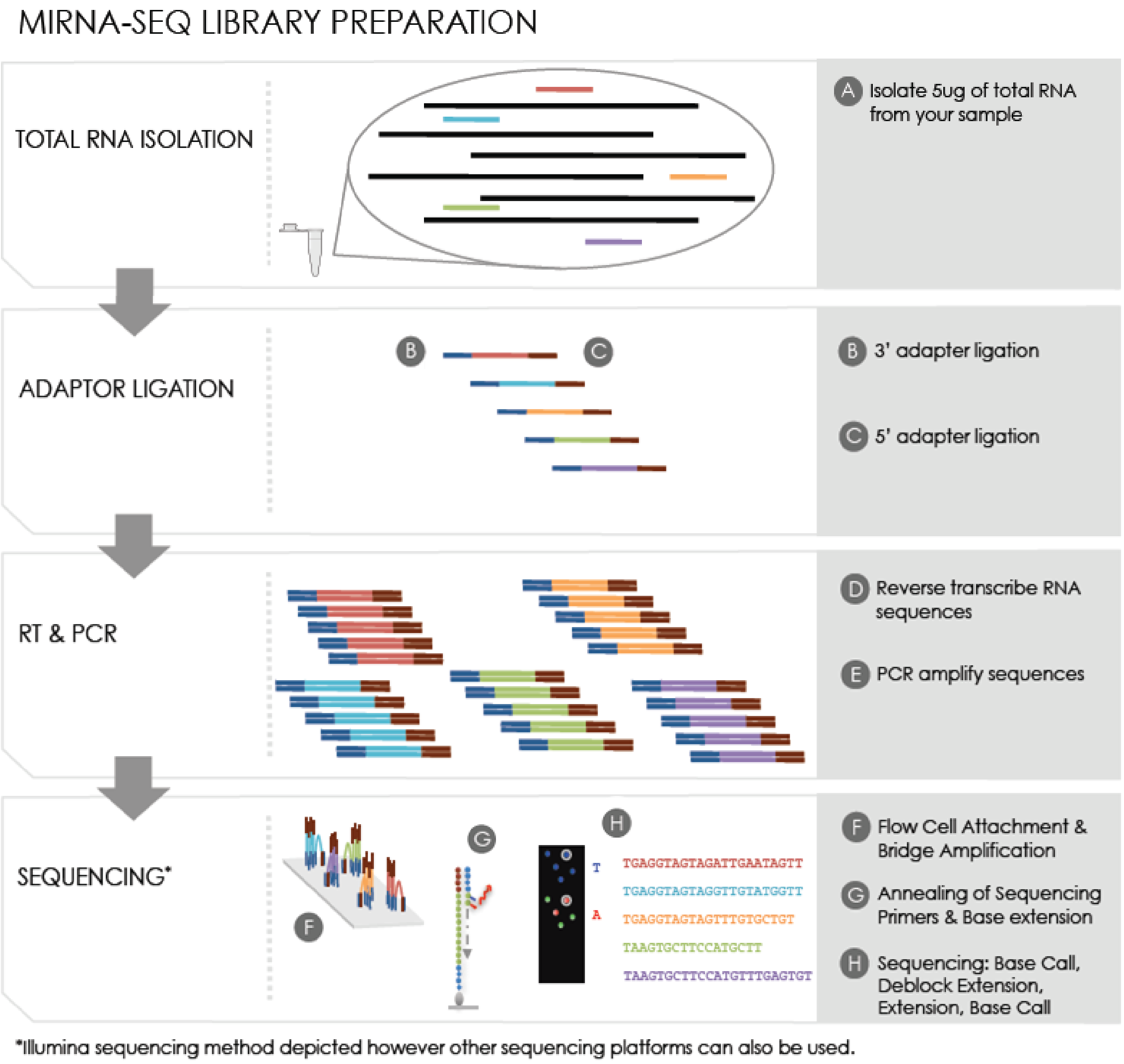

Figure 1. RNA Sequencing Experiment Workflow

REFERENCE datasheet_NEB_small_RNA_Library_Prep.pdf

1) Isolate the total RNA from a sample of interest using an isothiocyanate/phenol/chloroform (GITC/phenol) method or a commercial product such as Trizol (Invitrogen) reagent.

2) The sequencing adapters are ligated to the RNA molecules, which act as primer binding sites during reverse transcription and PCR amplification. Adenylated single strand DNA 3' adapter followed by a 5' adapter is ligated to the small RNAs using a ligating enzyme such as T4 RNA ligase 2. The adapters are also designed to capture small RNAs with 5' phosphate groups which is characteristic of microRNAs, rather than RNA degradation products with 5' hydroxyl group.

3) miRNA fragments with ligated adapters are converted to cDNA fragments which are later used in the sequencing reaction. There are many commercial kits using some form of reverse transcriptase in order to carry out this step. PCR is then performed to amplify the pool of cDNA sequences.

4) The amplified cDNA fragments are run on agarose gel and the band containing the molecules corresponding to the miRNA fragments with ligated adapters are cut out for subsequent sequencing (library size is ranged from 145 bp to 160 bp)

5) cDNA fragments are sequenced by the read length using sequence by synthesis method on the illumina platform.

2. Analysis Methods and Workflow

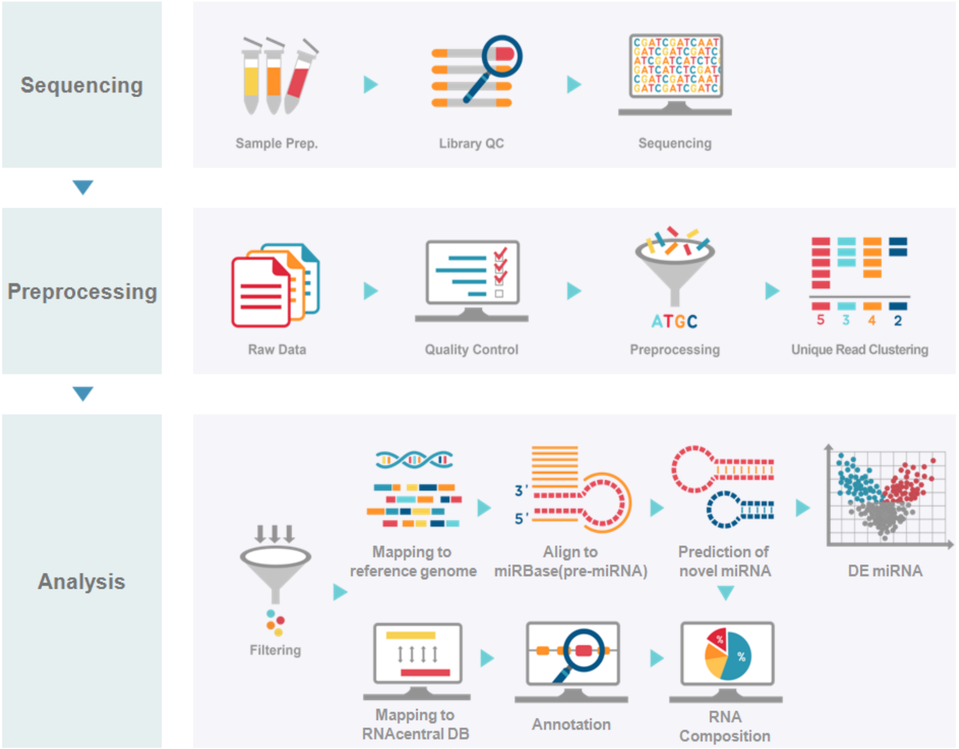

Figure 2. Analysis Workflow

The objectives of miRNA analysis are divided into four following contents.

- Quantification of miRNA expression
- Filtering differentially expressed miRNA
- Prediction of known / novel miRNAs
- Classification by categories of smRNA

1) After sequencing, the raw sequence reads are filtered based on quality. The adapter sequences are also trimmed off the raw sequence reads.
2) Both the trimmed reads and non-adapter reads as processed reads are used, to do analyzing long target (>= 50bp).
3) The processed reads are gathered forming a unique cluster. This cluster contains reads that are 100% match to the sequence identity as well as read length. The cluster is given its temporary cluster ID and the number of reads it holds.
4) In order to eliminate the effect of large amounts of rRNA from this study, the read was aligned to the rRNA sequence and have them removed.
5) rRNA removed reads are sequentially aligned to reference genome, miRBase v22.1 and non-coding RNA database, RNAcentral 14.0 to classify known miRNAs and other type of RNA such as tRNA, snRNA, snoRNA etc. Novel miRNA prediction is performed by miRDeep2.
6) The read counts for each miRNA are extracted from mapped miRNAs to report the abundance of each miRNA.

Humanizing Genomics macrogen

7) (Optional) Differentially expressed miRNAs are determined through comparing across conditions each miRNA using statistical methods.

3. Summary of Data Production

3. 1. Raw Data Statistics

(Refer to Path: result_smRNA/Analysis_statistics/FASTQ_stats.txt)

The total number of bases, reads, GC (%), Q20 (%), Q30 (%) are calculated for 3 samples. For example, in Control_Evs, 32,952,473 reads are produced, and total read bases are 3.3Gbp. The GC content (%) is 53.31% and Q30 is 79.46%.

Table 1. Raw data stats

Sample ID	Total read bases*	Total reads	GC (%)	Q20 (%)	Q30 (%)
Control_Evs	3,328,199,773	32,952,473	53.31	88.17	79.46
High_Hz_Evs	3,745,532,783	37,084,483	51.5	93.6	85.81
Low_Hz_Evs	3,336,236,040	33,032,040	52.77	95.42	89.87

(* Total read bases = Total reads x Read length)

- Total read bases: Total number of bases sequenced
- Total reads: Total number of reads
- GC (%): GC content
- Q20 (%): Ratio of bases that have phred quality score greater than or equal to 20
- Q30 (%): Ratio of bases that have phred quality score greater than or equal to 30

3. 2. Average Base Quality at Each Cycle

(Refer to Path: result_smRNA/Analysis_statistics/rawData_FASTQC/A_fastqc/)

The quality of produced data is determined by the phred quality score at each cycle. Box plot containing the average quality at each cycle is created with FastQC.

The x-axis shows number of cycles and y-axis shows phred quality score. Phred quality score 20 means 99% accuracy and reads over score of 20 are accepted as good quality.

Figure 3 shows average base quality at each cycle.

LINK http://www.bioinformatics.babraham.ac.uk/projects/fastqc

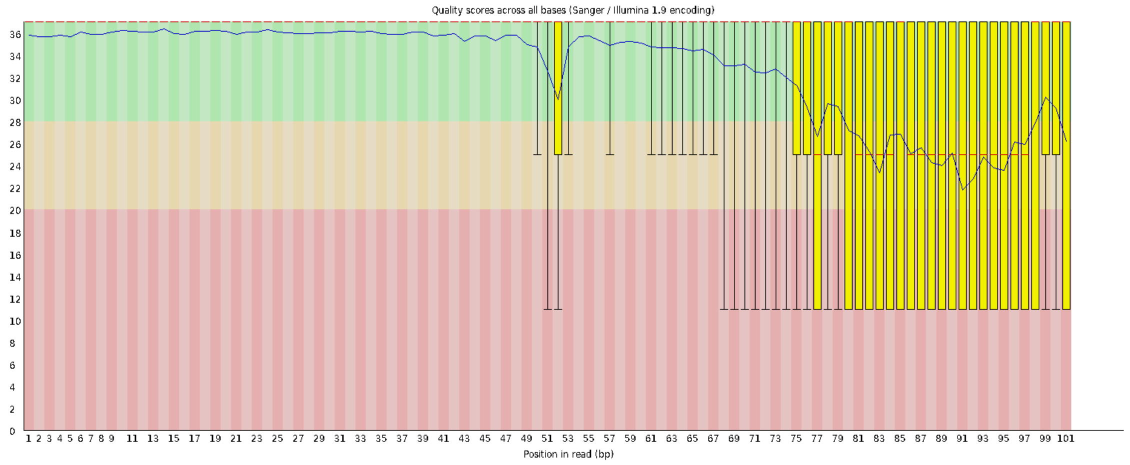

Figure 3. Read quality at each cycle of Control_Evs (read1)

- Yellow box: Interquartile range (25-75%) of phred score at each cycle
- Red line: Median of phred score at each cycle
- Blue line: Average of phred score at each cycle
- Green background: Good quality
- Orange background: Acceptable quality
- Red background: Bad quality

3. 3. Trimming Data Statistics

(Refer to Path: result_smRNA/Analysis_statistics/FASTQ_stats.txt)

The reads start at the first base after the 5' sequencing adapter and in Illumina sequencing typically end after 51 bp (in case of sequencing 51 bp SE). As mature miRNAs are normally up to 25 bp in length, the reads will contain part of 3' adapter sequence that has to be removed.

Sequenced reads are classified as trimmed reads, non-adapter reads, short reads and low quality reads according to these criteria. Adapter trimming process is done to eliminate the adapter sequences that exist in the read using Cutadapt. If a read matches at least first 5 bp of 3' adapter sequence, it is regarded as an adapter sequence, and then trimmed from the read. Trimmed reads should be at the minimum of 18 bp in order to be considered reliable for analysis. Afterwards, 3' end of each read is quality trimmed with -q 10 in Cutadapt. In addition, trimmed or nonAdapter read with 'N' base is regarded as low quality read and filtered.

Here is the definition of four read types described above.

- Trimmed Read: Read that is removed adapter sequences
- nonAdapter Read: Read that has not adapter sequences
- Short Read: Read with below 17bp in read length after adapter trimming
- Low Quality Read: Read with one or more 'N' base in trimmed or nonAdapter read

In case of analyzing short target (<50bp), the trimmed reads as processed reads are used. Otherwise, in case of analyzing long target (>= 50bp), both the trimmed reads and non-adapter reads as processed reads are used.

In this analysis, we analyzed both short target and long target to see comprehensive miRNA profiles.

For example, a total of 32,952,473 reads were produced from the Control_Evs sample, of which 29,794,947 reads included the adapter and the read length after trim was 18bp or more. 173,823 reads did not include the adapter, and 2,930,506 reads were shorter than 18bp after adapter trim.
For the analysis of long targets, 29,968,770 readings are used in the subsequent analysis, including trimmed reads and nonAdapter reads.

Table 2 - 4 show statistics of trimmed, nonAdapter and short FASTQ.

Table 2. Trimming Data Stats

Sample ID	Total read bases	Total reads	GC(%)	Q20(%)	Q30(%)
Control_Evs	865,776,541	29,794,947	53.97	98.24	94.17
High_Hz_Evs	1,099,732,302	36,712,000	53.16	98.28	94.17
Low_Hz_Evs	969,967,777	32,642,608	52.96	98.34	94.75

Table 3. nonAdapter Data Stats

Sample ID	Total read bases	Total reads	GC(%)	Q20(%)	Q30(%)
Control_Evs	17,556,123	173,823	53.01	74.45	61.36
High_Hz_Evs	10,582,679	104,779	42.02	71.89	56.74
Low_Hz_Evs	9,084,344	89,944	42.58	72.08	56.96

Table 4. Short Data Stats

Sample ID	Total read bases	Total reads	GC(%)	Q20(%)	Q30(%)
Control_Evs	29,865,144	2,930,506	59.74	97.94	94.13
High_Hz_Evs	2,168,662	202,140	64.18	98.1	94.44
Low_Hz_Evs	2,965,039	241,593	64.05	97.96	94.22

- Total read bases : Total number of read bases after trimming
- Total reads : Total number of reads after trimming
- GC(%) : GC Content
- Q20(%) : Ratio of bases that have phred quality score greater than or equal to 20
- Q30(%) : Ratio of bases that have phred quality score greater than or equal to 30

Table 5 and Figure 4 represents counts for trimmed reads, non-adapter reads, short reads and low quality reads by each sample.

Table 5. Summary of read preprocessing

Sample	Total Read Count	Trimmed Read Count	nonAdapter Read Count	Short Read Count	Low Quality Read Count
Control_Evs	32,952,473	29,794,947 (90.42%)	173,823 (0.53%)	2,930,506 (8.89%)	53,197 (0.16%)
High_Hz_Evs	37,084,483	36,712,000 (99.0%)	104,779 (0.28%)	202,140 (0.55%)	65,564 (0.18%)
Low_Hz_Evs	33,032,040	32,642,608 (98.82%)	89,944 (0.27%)	241,593 (0.73%)	57,895 (0.18%)

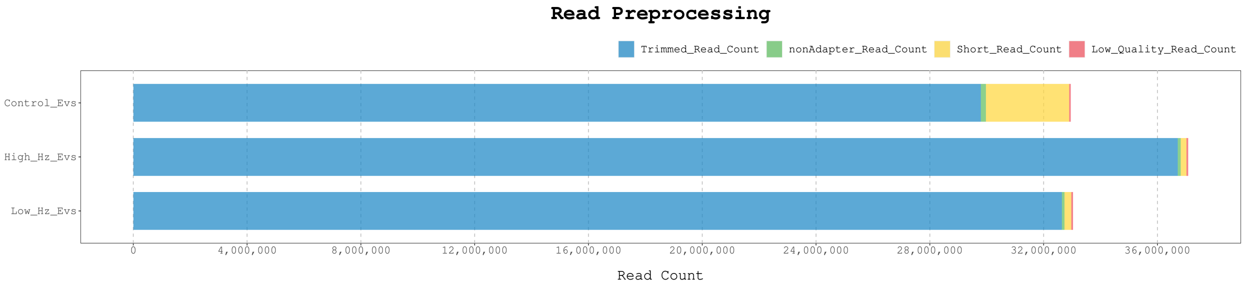

Figure 4. Summary of read preprocessing

3. 4. Average Base Quality at Each Cycle after Trimming

(Refer to Path: result_smRNA/Analysis_statistics/processedData_FASTQC/A_fastqc/)

Figure 5 shows average base quality at each cycle after trimming.

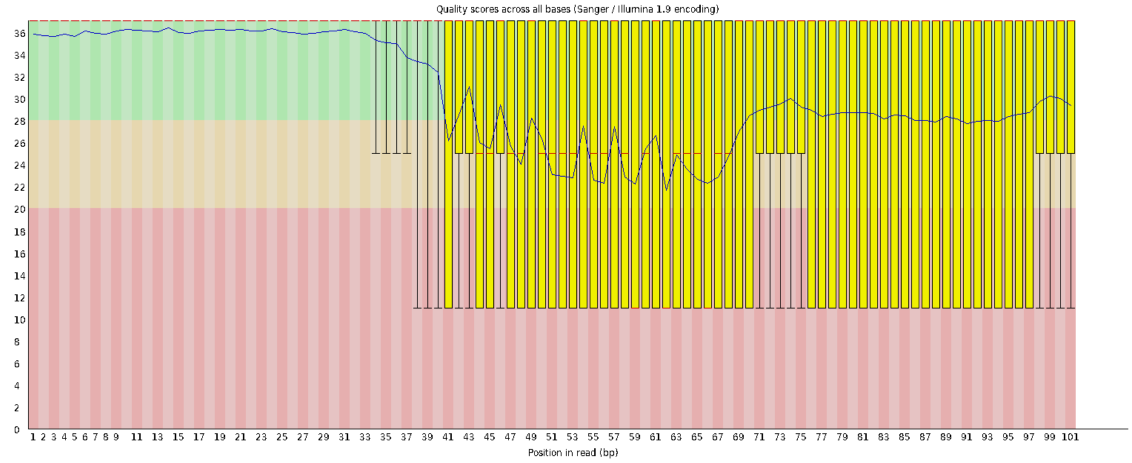

- Yellow box: Interquartile range (25-75%) of phred score at each cycle
- Red line: Median of phred score at each cycle
- Blue line: Average of phred score at each cycle
- Green background: Good quality
- Orange background: Acceptable quality
- Red background: Bad quality

3. 5. Final processed reads after removing unwanted reads

Most of the RNA composition is usually known as rRNA. In order to eliminate the effect of large amounts of rRNA from this study, reads were aligned to the 45S pre-rRNA and mitochondrial rRNA of *Mus musculus* and have them removed.

Figure 6 represents the remaining reads (blue) after removing rRNA (red).

For example a total of 29,968,770 reads left after adapter trimming step from the Control_Evs sample, of which 6,689,613 reads did aligned to rRNA sequences. 23,279,157 reads finally remained in this filtering step. These reads were used in the following analysis step.

Table 6. Remaining reads after removing rRNA

Sample	Total Read Count	Remain Read Count	Filtered Read Count
Control_Evs	29,968,770	23,279,157 (77.68%)	6,689,613 (22.32%)
High_Hz_Evs	36,816,779	32,548,045 (88.41%)	4,268,734 (11.59%)
Low_Hz_Evs	32,732,552	28,012,539 (85.58%)	4,720,013 (14.42%)

- Sample: Sample ID
- Total Read Count: Total read count of each sample
- Remain Read Count: Remaining reads count after removing rRNA
- Filtered Read Count: Filter out read count after removing rRNA

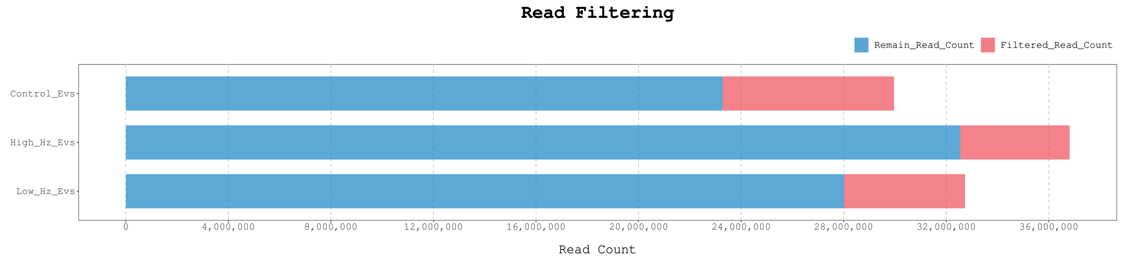

Figure 6. Summary of reads after removing rRNA

3. 6. Read length distribution of each sample

Figure 7 shows the read length distribution of each sample. Generally mature miRNAs are 20-25 nt in length.

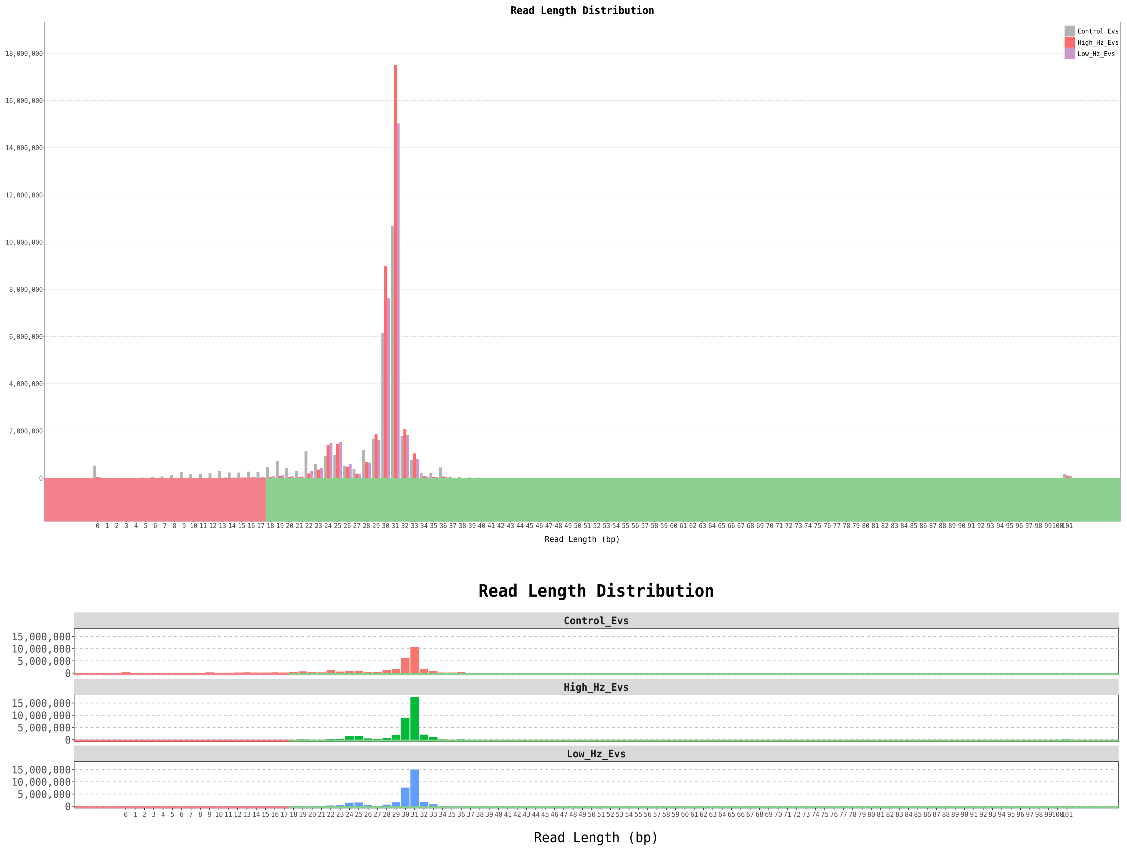

Figure 7. Length distribution of each sample

4. miRNA Analysis Result

4. 1. Summary of Small RNA Composition

For each sample, final processed reads are sequentially aligned to reference genome, miRBase v22.1 and non-coding RNA database (RNAcentral release 14.0) to classify known miRNAs and other types of RNA such as piRNA, tRNA, snRNA, snoRNA etc.

Genome mapping is processed by Bowtie and STAR using RSEM. Bowtie was subsequently used for miRDeep2 analysis using a genomic sequence. If it\'s necessary, the RSEM program can be used to align readings to transcript sequences using STAR.

Known/novel miRNA predicted by miRDeep2 and other smRNAs matching RNAcentral were aligned using Bowtie (target smRNA, <50 nt) and Bowtie2 (target smRNA, >=50nt).

Figure 8 represents the smRNA composition of each sample, which means the ratio of smRNA class type (such as known miRNA, candidate miRNA, rRNA, tRNA, snRNA, snoRNA etc.) classified from processed reads.

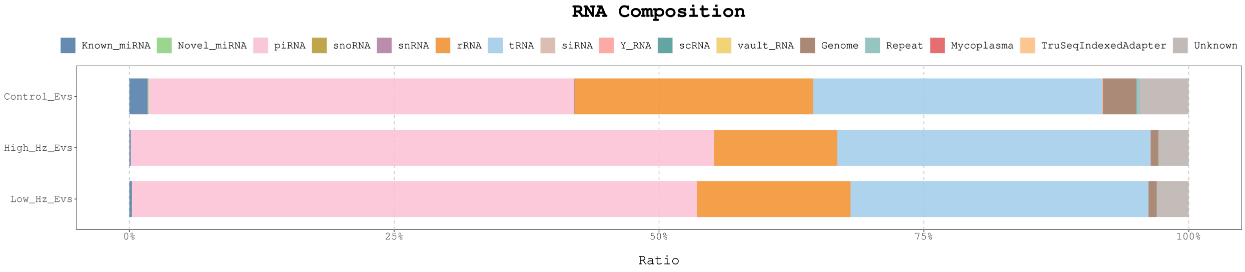

Figure 8. Summary of Small RNA Composition

4. 2. Quantification of Mature miRNA Abundance

Quantification step is divided into three procedures. At First, mature miRNA sequences of relevant species are aligned to precursor miRNA sequences from 22.1 (bowtie option : -f -v 0 -a --best --strata --norc). In the second step, unique clustered reads are aligned to precursor sequence (bowtie option : -f -v 1 -a --best --strata --norc). Bowtie aligner is used in the mapping steps. In the third step, we determine overlapping mature miRNA regions between two mapping results. Afterwards, the read count of mature miRNAs are extracted from the overlapping regions in the miRDeep2 Quantifier module. If a read is mapped to multiple mature miRNAs, the read counts are assigned equally to the abundance level of each mature miRNA. Table 7 shows the number of mapped reads to miRbase precursor, and the number of known mature miRNA with read count more than 1 for each sample.

Table 7. Mapped reads to miRBase precursor

Sample	Processed reads	Mapped reads	Known miRNA in Sample	Known miRNA in Species (miRBase v22.1)
Control_Evs	23,279,157	514,209 (2.21%)	324	1,978
High_Hz_Evs	32,548,045	51,015 (0.16%)	214	1,978
Low_Hz_Evs	28,012,539	79,031 (0.28%)	238	1,978

- Processed reads: Reads which were trimmed and removed unwanted sources from them
- Known miRNA in Sample: The number of known mature miRNA with read count more than 1 for each sample
- Known miRNA in Species: The number of known mature miRNA in miRBase database

Table 8 is an example of the expression profile of miRBase precursor for each sample in read count.

Table 9 shows the expression profile of known mature miRNA across samples. The expression profile of known mature miRNA is used to analyze differentially expressed miRNA (DE miRNA) later.

Table 8. Expression profile according to miRBase precursor (Example)

miRBase_Precursor_ID	hsa-mir-1246	hsa-mir-23a	hsa-mir-25	hsa-mir-191
Total_Read_Count	15191	2365	2176	2038
5p_miRNA_ID	hsa-miR-1246	hsa-miR-23a-5p	hsa-miR-25-5p	hsa-miR-191-5p
5p_Read_Counts	15191	1209	52	2036
3p_miRNA_ID		hsa-miR-23a-3p	hsa-miR-25-3p	hsa-miR-191-3p
3p_Read_Counts		1156	2121	2
Remaining_Reads	0	0	3	0
miRBase_5p_Sequence(s)	aauggauuuuuggagcagg	ugagguaguagguuguauaguu	gggguuccuggggaugggauuu	aggcggagacuugggcaauug
miRBase_3p_Sequence(s)		cuguacagccuccuagcuuucc	aucacauugccagggauuucc	cauugcacuugucucggucuga
miRBase_Precursor_Sequence	uguauccuugaauggauuuuuggagcaggaguggacaccugacccaaaggaaaucaauccauaggcuagcaau	agguugagguaguagguuguauaguuuagaauuacaucaagggagauaacuguacagccuccuagcuuuccu	ggccggcuggggguuccuggggaugggauuugcuuccugucacaaaucacauugccagggauuuccaaccgacc	ggccaguguugagaggcggagacuugggcaauugcuggacgcugcccugggcauugcacuugucucggucugacagugccggcc

- miRBase Precursor ID: Clicking this field will display a pdf of the structure and read signature of the miRNA.
- Total Read Count: The sum of read counts for the 5p and 3p miRNA
- 5p miRNA ID
- 5p Read Counts: The number of reads that mapped to the miRNA hairpin and are contained in the sequence covered by the 5p sequence, including 2 nts upstream and 5 nts downstream.
- 3p miRNA ID
- 3p Read Counts: The number of reads that mapped to the miRNA hairpin and are contained in the sequence covered by the 3p sequence, including 2 nts upstream and 5 nts downstream.
- Remaining Reads: The number of reads that did not map to any of the 5p or 3p miRNA
- miRBase 5p sequence(s): The 5p miRNA sequence(s)
- miRBase 3p sequence(s): The 3p miRNA sequence(s)
- miRBase Precursor sequence: The precursor miRNA sequence

[PDF description based on Mature miRNA]

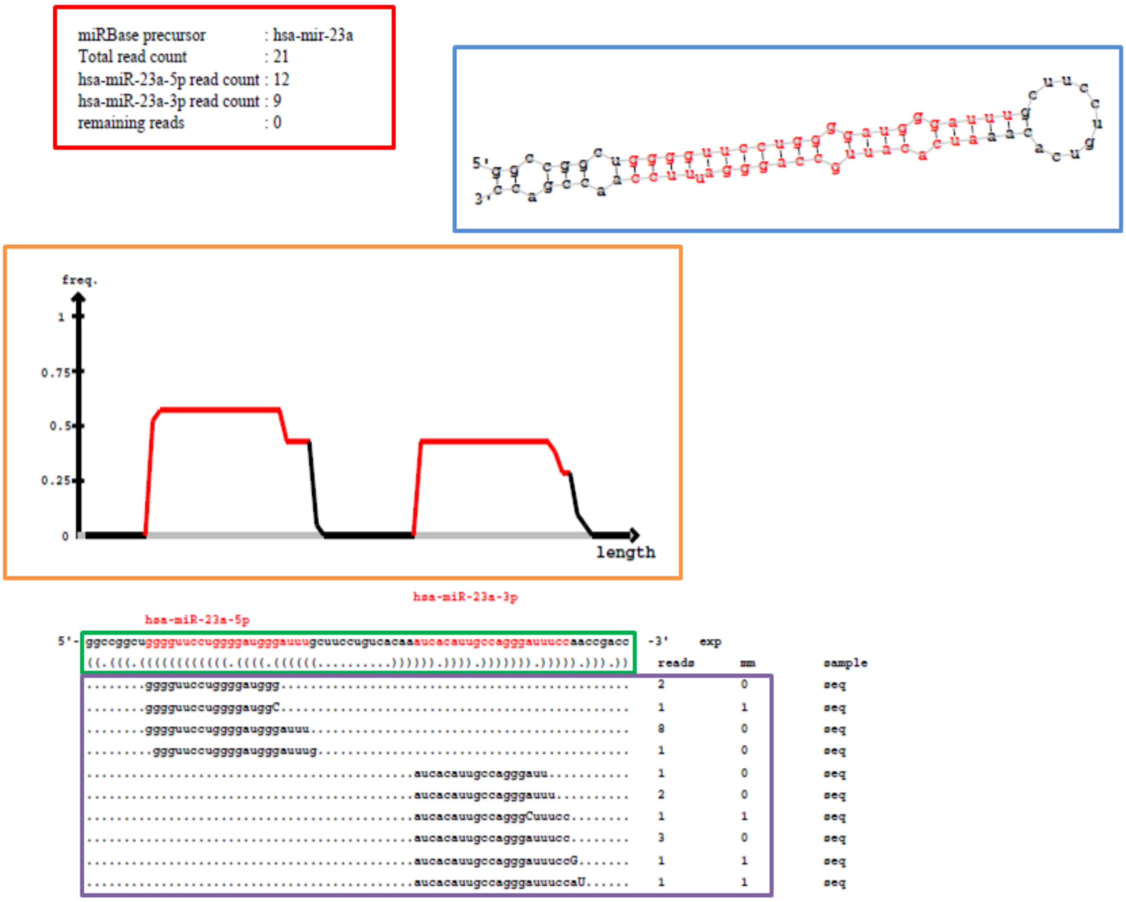

[Red Box]: Information box

- miRBase precursor: Precursor sequence ID of miRBase
- Total read count: Total read count of precursor
- 5p read counts: The number of reads that mapped to the miRNA hairpin and are contained in the sequence covered by the 5p sequence, including 2 nts upstream and 5 nts downstream
- 3p read counts: The number of reads that mapped to the miRNA hairpin and are contained in the sequence covered by the 3p sequence, including 2 nts upstream and 5 nts downstream
- remaining reads: The number of reads that did not map to any of the 5p or 3p sequences

[Blue Box]: Secondary structure box; (Figure)

- Red character: Mature miRNA region

[Orange Box]: Read frequency distribution

- Red line: Mature miRNA region

[Green Box]: Secondary structure box; (Text)

- First line: Genomic sequence (Red character: mature miRNA region)
- Second line: Secondary structure ("..."; Wobble, have not bond, "(", ")"; have bond)

[Purple Box]: Read Box

- Column 1: Read sequence (Upper case: mismatch sequence)
- Column 2: Read Count
- Column 3: Mismatch count

Table 9. Expression profile according to known mature miRNA (Example)

Mature_ID	hsa-let-7a-2-3p	hsa-let-7a-3p	hsa-let-7a-5p	hsa-let-7b-3p	hsa-let-7c-5p	hsa-let-7d-5p
Mature_Accession	MIMAT0010195	MIMAT0004481	MIMAT0000062	MIMAT0004482	MIMAT0000064	MIMAT0000065
Hairpin_ID	hsa-let-7a-2	hsa-let-7a-1, hsa-let-7a-3	hsa-let-7a-1, hsa-let-7a-2, hsa-let-7a-3	hsa-let-7b	hsa-let-7c	hsa-let-7d
Hairpin_Accession	MI0000061	MI0000060, MI0000062	MI0000060, MI0000061, MI0000062	MI0000063	MI0000064	MI0000065
miFAM_ID	let-7	let-7	let-7	let-7	let-7	let-7
miFAM_Accession	MIPF0000002	MIPF0000002	MIPF0000002	MIPF0000002	MIPF0000002	MIPF0000002
miRBase_Link	hsa-let-7a-2-3p	hsa-let-7a-3p	hsa-let-7a-5p	hsa-let-7b-3p	hsa-let-7c-5p	hsa-let-7d-5p
ENTREZGENE_Link	406882	406881	406881	406884	406885	406886
RFAM_Link	RF00027	RF00027	RF00027	RF00027	RF00027	RF00027
HGNC_Link	31477	31476	31476	31479	31480	31481
AM_RPM	0	35.197015	51669.21845	123.189554	11738.2046	6300.265737
BM_RPM	0	330.654552	67309.76581	244.396843	26754.26616	4600.411162
AM_Read_Count	0	2	2936	7	667	358
BM_Read_Count	0	23	4682	17	1861	320

- Mature ID: Mature miRNA ID
- Mature Accession: Mature miRNA accession ID of miRBase
- Hairpin ID: Hairpin (Precursor) ID
- Hairpin Accession: Hairpin accession ID of miRBase
- miFAM ID: miRNA gene family ID
- miRBase Link: miRBase ID (click to miRBase)
- ENTREZGENE Link: ENTREZGENE accession (click to NCBI)
- HGNC Link: HGNC accession (click to HUGO Gene Nomenclature Committee) if species is Homo sapiens, this column displays.
- MGI Link: MGI accession (click to Mouse Genome Information) if species is Mus musculus, this column displays.
- RFAM Link: RFAM accession (click to RNA families database)
- [Sample Name] RPM : Read per million of sample called "Sample Name"
- [Sample Name] Read Count: Read count of the sample called "Sample Name"

4. 3. Prediction of known/novel miRNA

To predict the known and novel miRNA, unique clustered reads are aligned against reference genome and precursor miRNAs separately. Novel microRNAs are predicted from mature, star and loop sequence according to the RNAfold algorithm using miRDeep2. The RNAfold function uses the nearest-neighbor thermodynamic model to predict the minimum free-energy secondary structure of an RNA sequence. RNAfold-generated graphic contains the actual in silico-folded hairpin, with the number of reads for each part of the hairpin, score for minimum free energy, score for randfold, and score for conserved seed sequence. In addition to detecting known and novel miRNAs, miRDeep2 estimates their abundance. Table 10 shows the mapping statistics to mm10 reference genome with Bowtie.

Table 10. Mapped reads to reference genome

Sample	Total reads	Mapped reads	Unmapped reads
Control_Evs	23,279,157	2,282,184 (9.8%)	20,996,973 (90.2%)
High_Hz_Evs	32,548,045	1,552,034 (4.77%)	30,996,011 (95.23%)
Low_Hz_Evs	28,012,539	1,460,872 (5.22%)	26,551,667 (94.78%)

Table 11 - 14 and Genome-based PDF refer to result_smRNA_excel/miRDeep2_Result/[Sample_name]/[Sample_Name]_GenomeBased_result.xlsx

Table 11 shows an example of the summary result of predicted known/novel miRNAs by miRDeep2 at score cut-offs from -10 to 10.

Table 11. Survey of miRDeep2 performance at score cut-offs from -10 to 10

miRDeep2 score	10	9	8
novel miRNAs reported by miRDeep2	10	10	10
novel miRNAs, estimated false positives	8 +/- 3	8 +/- 3	8 +/- 3
novel miRNAs, estimated true positives	2 +/- 2 (24 +/- 21%)	2 +/- 2 (23 +/- 21%)	2 +/- 2 (21 +/- 20%)
known miRNAs in species	2588	2588	2588
known miRNAs in data	230	230	230
known miRNAs detected by miRDeep2	32 (14%)	34 (15%)	35 (15%)
estimated signal-to-noise	3.3	3.3	3.3
excision gearing	2	2	2

- miRDeep2 score: For details on how the log-odds score is calculated, see Friedlander et al., Nature Biotechnology, 2008.
- predicted by miRDeep2: Novel miRNA hairpins are defined by not having any of the reference mature miRNAs mapping perfectly (full length, no mismatches). The numbers show how many novel miRNA hairpins have a score equal to or exceeding the cut-off.
- estimated false positives: Number of false positive miRNA hairpins predicted at this cut-off, as estimated by the miRDeep2 controls (see Friedlander et al., Nature Biotechnology, 2008). Mean and standard deviation are estimated from 100 rounds of permuted controls.
- estimated true positives: The number of true positive miRNA hairpins are estimated as t = total novel miRNAs - false positive novel miRNAs. The percentage of the predicted novel miRNAs that is estimated to be true positives is calculated as p = t/total novel miRNAs. The number of false positives are estimated from 100 rounds of permuted controls. In each of the 100 rounds, t and p are calculated, generating mean and standard deviation of t and p. The variable p can be used as an estimation of miRDeep2 positive predictive value at the score cut-off.
- in species: Number of reference mature miRNAs for that species given as input to miRDeep2.
- in data: Number of reference mature miRNAs for that species that map perfectly (full length, no mismatches) to one or more of precursor candidates that have been excised from the genome by miRDeep2.
- detected by miRDeep2 : Number of reference mature miRNAs for that species that map perfectly (full length, no mismatches) to one or more of predicted miRNA hairpins that have a score equal to or exceeding the cut-off. The percentage of reference mature miRNAs in data that is detected by miRDeep2 is calculated as s = reference mature miRNAs detected / reference mature miRNAs in data. s can be used as an estimation of miRDeep2 sensitivity at the score cut-off.
- estimated signal-to-noise: For the given score cut-off, the signal-to-noise ratio is estimated as r = total miRNA hairpins reported/mean estimated false positive miRNA hairpins over 100 rounds of permuted controls.
- excision gearing: This is the minimum read stack height required for excising a potential miRNA precursor from the genome in this analysis.

There are three prediction results by miRDee2:

- novel miRNAs predicted by miRDeep2
- mature miRBase miRNAs detected by miRDeep2
- mature miRBase miRNAs not detected by miRDeep2

Table 12. novel miRNAs predicted by miRDeep2 (Example)

provisional id	chrX_33645	chr6_11385	chr13_22777
miRDeep2 score	258.3	258.2	156.4
estimated probability that the miRNA candidate is a true positive	24 +/- 21%	24 +/- 21%	24 +/- 21%
total read count	507	506	306
mature read count	504	504	278
loop read count	0	0	0
star read count	3	2	28
significant randfold p-value	yes	yes	yes
UCSC browser	blat	blat	blat
consensus mature sequence	ggaggcggagguugcagugagc	ggaggcggagguugcagugagc	ggaggcggagguugcagugagc
consensus star sequence	ugccauugcacuccagccugggcg	gccauugcacuccagccug	cacugcacuccagccugggc
consensus precursor sequence	ggaggcggagguugcagugagccaagauugugccauugcacuccagccugggcg	ggaggcggagguugcagugagccaagauugcgccauugcacuccagccug	ggaggcggagguugcagugagccaagauugcaccacugcacuccagccugggc
precursor coordinate	chrX:119035594..119035648:+	chr6:35484229..35484279:+	chr13:31066230..31066283:+

- provisional id: A provisional miRNA name assigned by miRDeep2. The first part of the id designates the chromosome or genome contig on which the miRNA gene is located. The second part is a running number that is added to avoid identical ids. The running number is incremented by one for each potential miRNA precursor that is excised from the genome. Clicking this field will display a pdf of the structure, read signature and score breakdown of the reported miRNA.
- miRDeep2 score: The log-odds score assigned to the hairpin.
- estimated probability that the miRNA candidate is a true positive: The estimated probability that a predicted novel miRNA with a score of this or higher is a true positive. To see exactly how this probability is estimated, mouse over the 'novel miRNAs, true positives' in the table at the top of the webpage.
- total read count: The sum of read counts for the predicted mature, loop and star miRNAs.
- mature read count: The number of reads that mapped to the predicted miRNA hairpin and are contained in the sequence covered by the predicted mature miRNA, including 2 nts upstream and 5 nts downstream.
- loop read count: The number of reads that mapped to the predicted miRNA hairpin and are contained in the sequence covered by the predicted miRNA loop, including 2 nts upstream and 5 nts downstream.
- star read count: The number of reads that mapped to the predicted miRNA hairpin and are contained in the sequence covered by the predicted star miRNA, including 2 nts upstream and 5 nts downstream.
- significant randfold p-value: If the estimated randfold p-value of the excised potential miRNA hairpin is lower or equal to than 0.05 (see Bonnet et al., Bioinformatics, 2004).
- UCSC browser: If a species name was put into miRDeep2, then clicking this field will initiate

a UCSC blat search of the consensus precursor sequence against the reference genome.

- consensus mature sequence: The consensus mature miRNA sequence as inferred from the deep sequencing reads.
- consensus star sequence: The consensus star miRNA sequence as inferred from the deep sequencing reads.
- consensus precursor sequence: The consensus precursor miRNA sequence as inferred from the deep sequencing reads. Note that this is the inferred Drosha hairpin product, and therefore does not include substantial flanking genomic sequence as does most miRBase precursors.
- precursor coordinate: The given precursor coordinates refer to absolute position in the mapped reference sequence.

Table 13. mature miRBase miRNAs detected by miRDeep2 (Example)

tag id	chr17_27374	chr7_14462	chr22_32774
miRDeep2 score	1580.6	174.5	122.3
estimated probability that the miRNA is a true positive	24 +/- 21%	24 +/- 21%	24 +/- 21%
predicted mature seq. in accordance with miRBase mature seq.	TRUE	STAR	TRUE
total read count	3097	347	236
mature read count	3080	346	233
loop read count	0	0	0
star read count	17	1	3
significant randfold p-value	yes	no	yes
mature miRBase miRNA	hsa-miR-423-5p	hsa-miR-25-5p	hsa-let-7b-5p
UCSC browser	blat	blat	blat
consensus mature sequence	ugaggggcagagagcgagacuuu	cauugcacuugucucggucug	ugagguaguagguugugugguu
consensus star sequence	agcucggucugaggccccucagu	aggcggagacuugggcaauug	cuauacaaccuacugccuuccc
consensus precursor sequence	ugaggggcagagagcgagacuuu ucuauuuuccaaaagcucggucug aggccccucagu	aggcggagacuugggcaauugcug gacgcugcccugggcauugcacuu gucucggucug	ugagguaguagguugugugguu ucagggcagugauguugccccucg gaagauaacuauacaaccuacugcc uuccc
precursor coordinate	chr17:28444112..28444171:+	chr7:99691194..99691253:-	chr22:46509570..46509646:+

- tag id: A tag id assigned by miRDeep2. The first part of the id designates the chromosome or genome contig on which the miRNA gene is located. The second part is a running number that is added to avoid identical ids. The running number is incremented by one for each potential miRNA precursor that is excised from the genome. Clicking this field will display a pdf of the structure, read signature and score breakdown of the miRNA.
- miRDeep2 score: The log-odds score assigned to the hairpin by miRDeep2
- estimated probability that the miRNA is a true positive: The estimated probability that a predicted miRNA with a score of this or higher is a true positive. For miRBase miRNAs, this reflects the support that the data at hand lends to the miRNA.
- predicted mature seq. in accordance with miRBase mature seq.: If the predicted miRDeep2 sequence overlaps with the miRBase annotated mature sequence than this is indicated by 'TRUE'. If the predicted miRDeep2 star sequence overlaps with the miRBase annotated mature sequence this is inidicated by 'STAR'.
- total read count: The sum of read counts for the mature, loop and star miRNAs.
- mature read count: The number of reads that mapped to the miRNA hairpin and are contained in the sequence covered by the consensus mature miRNA, including 2 nts upstream and 5 nts downstream.
- loop read count: The number of reads that mapped to the miRNA hairpin and are contained in the sequence covered by the consensus miRNA loop, including 2 nts upstream and 5 nts downstream.
- star read count: The number of reads that mapped to the miRNA hairpin and are contained in the sequence covered by the consensus star miRNA, including 2 nts upstream and 5 nts downstream.
- significant randfold p-value: If the estimated randfold p-value of the miRNA hairpin is lower or equal to than 0.05 (see Bonnet et al., Bioinformatics, 2004).
- mature miRBase miRNA: The ids of any reference mature miRNAs for the species that

mapped perfectly (full length, no mismatches) to the reported miRNA hairpin. If this is the case, the reported miRNA hairpin is assigned as a known miRNA. If not, it is assigned as a novel miRNA. If more than one reference mature miRNA maps to the miRNA hairpin, then only the id of the reference miRBase miRNA that matches the predicted mature sequence is output.

- UCSC browser: If a species name was put into miRDeep2, then clicking this field will initiate a UCSC blat search of the consensus precursor sequence against the reference genome.
- consensus mature sequence: The consensus mature miRNA sequence as inferred from the deep sequencing reads.
- consensus star sequence: The consensus star miRNA sequence as inferred from the deep sequencing reads.
- consensus precursor sequence: The consensus precursor miRNA sequence as inferred from the deep sequencing reads. Note that this is the inferred Drosha hairpin product, and therefore does not include substantial flanking genomic sequence as does most miRBase precursors.
- precursor coordinate: The given precursor coordinates refer to absolute position in the mapped reference sequence

Table 14. mature miRBase miRNAs not detected by miRDeep2 (Example)

miRBase_Precursor_ID	hsa-mir-1290	hsa-mir-6126	hsa-mir-619
Total_Read_Count	4475	2078	401
5p_Read_Counts	0	2078	222
3p_Read_Counts	4475	0	0
Remaining_Reads	0	0	179
miRBase_5p_Sequence(s)	-	gugaaggcccggcggaga	gcugggauuacaggcaugagcc
miRBase_3p_Sequence(s)	uggauuuuuggaucaggga	-	gaccuggacauguuugugcccagu
miRBase_Precursor_Sequence	gagcgucacguugacacucaaaaaguuucagauuuuggaacauuucggauuuuggauuuuuggaucagggaugcucaa	agccugugggaaagagaagagcagggcagggugaaggcccggcggagacacucugcccaccccacacccugccuaugggccacacagcu	cgcccaccucagccucccaaaaugcugggauuacaggcaugagccacugcggucgaccaugaccuggacauguuugugcccaguacugucaguuugcag

- miRBase precursor id: Clicking this field will display a pdf of the structure and read signature of the miRNA.
- total read count: The sum of read counts for the mature and star miRNAs.
- 5p read counts: The number of reads that mapped to the miRNA hairpin and are contained in the sequence covered by the 5p sequence, including 2 nts upstream and 5 nts downstream.
- 3p read counts: The number of reads that mapped to the miRNA hairpin and are contained in the sequence covered by the 3p sequence, including 2 nts upstream and 5 nts downstream.
- remaining reads: The number of reads that did not map to any of the mature and star sequences
- miRBase 5p sequence(s): The 5p miRNA sequence(s)
- miRBase 3p sequence(s): The 3p miRNA sequence(s)

- miRBase precursor sequence: This is the precursor miRNA sequence

[PDF description based on predicted precursor miRNA]

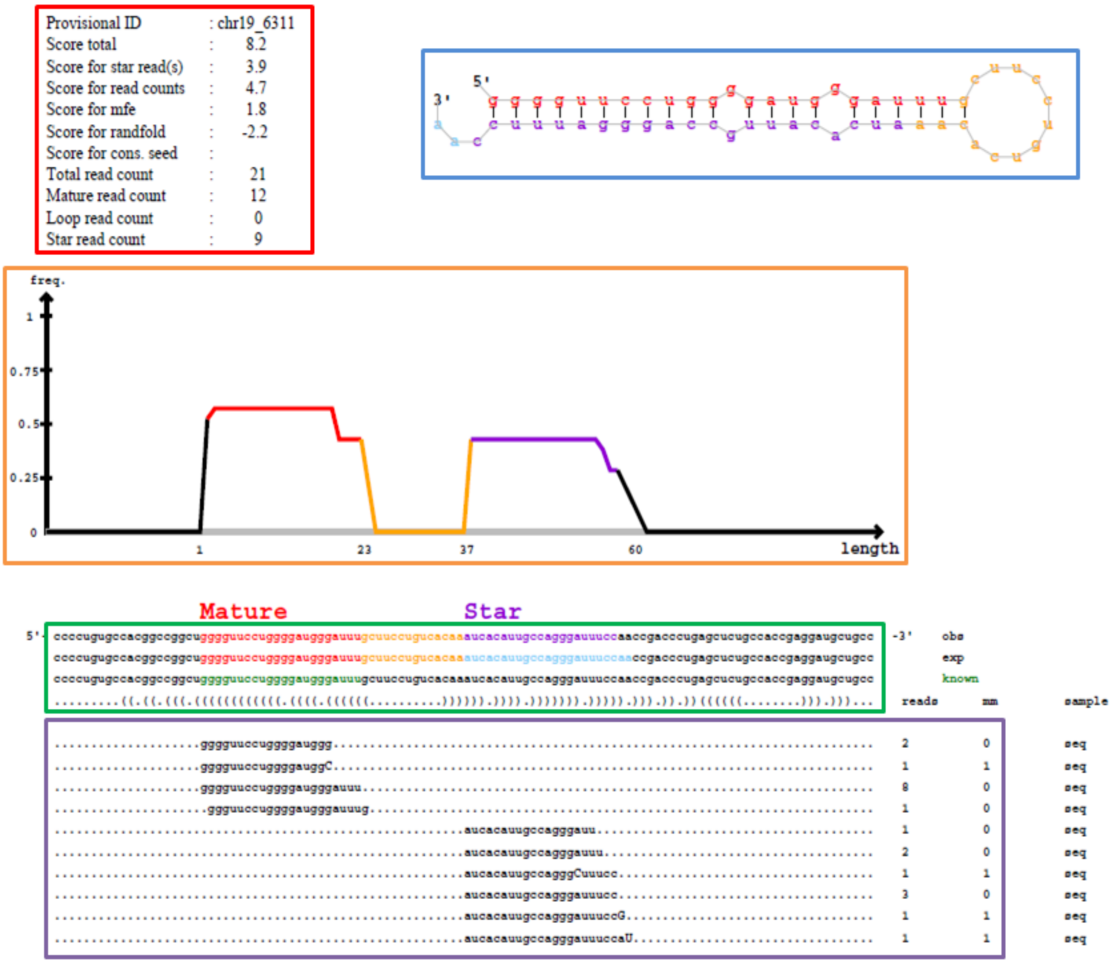

[Red Box]: Information box (score algorithm in miRDeep2_core_algorithm.pl)

- Provisional ID: A provisional miRNA name assigned by miRDeep2.
- Score total: Sum of the scores below
- Score for star read(s): Scores for the majority of potential star reads fall as expected from Dicer processing (true: 3, false: -1.3)
- Score for read counts: Scores for the count of reads that map to the potential precursor
- Score for mfe: Scores for the minimal free energy of the potential precursor
- Score for randfold: Scores for the Randfold folding test (true: 1.6, false: -2.2)
- Score for cons. Seed: scoring for conserved seed (true: +3, false: -0.6)
- Total read count: The sum of read counts for the mature, loop and star miRNAs.
- Mature read count: The number of reads that mapped to the miRNA hairpin and are contained in the sequence covered by the consensus mature miRNA, including 2 nts upstream and 5 nts downstream.
- Loop read count: The number of reads that mapped to the miRNA hairpin and are contained in the sequence covered by the consensus miRNA loop, including 2 nts upstream and 5 nts downstream.
- Star read count: The number of reads that mapped to the miRNA hairpin and are contained

in the sequence covered by the consensus star miRNA, including 2 nts upstream and 5 nts downstream.

[Blue Box]: Secondary structure box; (Figure)

- Red character: Mature miRNA region
- Purple character: Star region
- Yellow character: Loop region

[Orange Box]: Read frequency distribution

- Red line: Mature miRNA region
- Purple character: Star region
- Yellow character: Loop region

[Green Box]: Secondary structure box; (Text)

- "exp" line: mature, loop, and star region expected by miRDeep2 algorithm (Red Character: mature miRNA region, Purple character: star region, Yellow character: loop region)
- "obs" line: mature, loop, and star region observed by read align result (Red Character: mature miRNA region, Purple character: star region, Yellow character: loop region)
- "known" line: The region that matches the known mature miRNA of an already miRBase (Green character)
- Last line: Secondary structure ("…"; Wobble, have not bond, "(", ")"; have bond)

[Purple Box]: Read Box

- Column 1: Read sequence (Upper case: mismatch sequence)
- Column 2: Read Count
- Column 3: Mismatch count

In order to compare the predicted miRNA information for each sample, novel miRNAs overlapping 70% or more in the same strand were assumed to be the same miRNA.

Table 15, 16 shows an example of the summary of merged novel precursor miRNAs. Refer to Expression_Profile.mmu.10090.RNAcentral.xlsx - Novel.precursor.Exp, Novel.precursor.Info sheet.

Table 17, 18 shows an example of the summary of merged novel mature miRNAs. Refer to Expression_Profile.mmu.10090.RNAcentral.xlsx - Novel.mature.Exp, Novel.mature.Info sheet.

Table 15. Expression profile according to novel precursor miRNA (Example)

Precursor_ID	Chr	Start	End	Strand	Length	AM_Read_Count	BM_Read_Count
Precursor_1	chr1	248074635	248074715	-	81	5517	3238
Precursor_2	chr1	249115811	249115882	-	72	24	0
Precursor_3	chr2	3721463	3721549	+	87	78	0
Precursor_4	chr2	6985560	6985610	-	51	0	18
Precursor_5	chr2	7539768	7539831	+	64	24	0
Precursor_6	chr2	25564019	25564087	-	69	18	0
Precursor_7	chr2	26278302	26278348	-	47	415	0
Precursor_8	chr2	28508623	28508683	-	61	0	103
Precursor_9	chr2	38816200	38816241	+	42	277	0
Precursor_10	chr2	38830014	38830074	-	61	0	15

- Precursor ID: merged precursor miRNA ID
- Chr, Start, End, Strand: Genomic position of merged precursor miRNA
- Length: Length of merged precursor miRNA
- [Sample] Read Count: Read count of the sample called "Sample Name"

Table 16. Information according to novel precursor miRNA (Example)

Precursor_ID	Precursor_1	Precursor_2	Precursor_3	Precursor_4	Precursor_5	Precursor_6
Chr	chr1	chr1	chr2	chr2	chr2	chr2
Start	248074635	249115811	3721463	6985560	7539768	25564019
End	248074715	249115882	3721549	6985610	7539831	25564087
Strand	-	-	+	-	+	-
Length	81	72	87	51	64	69
Total_Count	8755	24	78	18	24	65
Total_Quality	4.5	2	0	2.3	0.2	7
AM_ID	chr1_4580|chr1:248074635-248074715:-	chr1_4581|chr1:249115811-249115882:-	chr2_4608|chr2:3721463-3721549:+	.	chr2_4626|chr2:7539768-7539831:+	chr2_6589|chr2:25564019-25564087:-
AM_Read_Count	5517	24	78	.	24	18
AM_Quality	2.2	2	0	.	0.2	3.5
BM_ID	chr1_4932|chr1:248074637-248074713:-	.	.	chr2_6887|chr2:6985560-6985610:-	.	.
BM_Read_Count	3238	.	.	18	.	.
BM_Quality	2.3	.	.	2.3	.	.

- Precursor ID: merged precursor miRNA ID
- Chr, Start, End, Strand: Genomic position of merged precursor miRNA
- Length: Length of merged precursor miRNA
- Total Count: Sum of read count for each sample

- Total Quality: Sum of quality for each sample
- [Sample] ID, Read Count, Quality: Novel precursor miRNA ID, Read Count, Quality of each sample

Table 17. Expression profile according to novel mature miRNA (Example)

Mature_ID	Chr	Start	End	Strand	Length	AM_Read_Count	BM_Read_Count
Mature_1	chr1	11423039	11423058	+	20	348	423
Mature_2	chr1	23150271	23150293	-	23	47	10
Mature_3	chr1	27320065	27320083	+	19	0	18453
Mature_4	chr1	27458337	27458359	+	23	0	425
Mature_5	chr1	31508166	31508187	+	22	208	0
Mature_6	chr1	33788507	33788526	-	20	12	8
Mature_7	chr1	38325303	38325321	+	19	0	38
Mature_8	chr1	39080606	39080624	-	19	514	0
Mature_9	chr1	42217030	42217048	+	19	26	19
Mature_10	chr1	248074635	248074653	-	19	5517	3238

- Precursor ID: merged mature miRNA ID
- Chr, Start, End, Strand: Genomic position of merged mature miRNA
- Length: Length of merged mature miRNA
- [Sample] Read Count: Read count of the sample called "Sample Name"

Table 18. Information according to novel mature miRNA (Example)

Mature_ID	Mature_1	Mature_2	Mature_3	Mature_4	Mature_5	Mature_6
Chr	chr1	chr1	chr1	chr1	chr1	chr1
Start	11423039	23150271	27320065	27458337	31508166	33788507
End	11423058	23150293	27320083	27458359	31508187	33788526
Strand	+	-	+	+	+	-
Length	20	23	19	23	22	20
Total_Count	771	57	18451	425	208	20
Total_Quality	3	2.1	2.9	0.8	0.4	1.9
AM_ID	chr1_559_mature|chr1:11423039-11423058:+	chr1_2858_mature|chr1:23150271-23150293:-	.	.	chr1_861_mature|chr1:31508166-31508187:+	chr1_3011_mature|chr1:33788507-33788526:-
AM_Read_Count	348	47	.	.	208	12
AM_Quality	1.5	1.5	.	.	0.4	1.9
BM_ID	chr1_599_mature|chr1:11423039-11423058:+	chr1_3050_mature|chr1:23150271-23150293:-	chr1_840_mature|chr1:27320065-27320083:+	chr1_843_mature|chr1:27458337-27458359:+	.	chr1_3211_mature|chr1:33788508-33788526:-
BM_Read_Count	423	10	18451	425	.	8
BM_Quality	1.5	0.6	2.9	0.8	.	0

- Precursor ID: merged mature miRNA ID
- Chr, Start, End, Strand: Genomic position of merged mature miRNA
- Length: Length of merged mature miRNA
- Total Count: Sum of read count for each sample
- Total Quality: Sum of quality for each sample
- [Sample] ID, Read Count, Quality: Novel mature miRNA ID, Read Count, Quality of each sample

4. 4. Small RNA Composition

There are various kinds of small RNAs in cells. In this analysis, we used RNAcentral 14.0 DB to investigate the composition of small RNA for each sample.

Final processed reads were aligned to small RNAs (50 nt or less; piRNA) of the DB using bowtie (option: -f -l 15 --norc -v 1 -a) and to small RNAs (50nt or greater; tRNA, snoRNA, etc.) of the DB using bowtie2 (option: -f -L 15 --norc -a) which assigned a result of 90% or more of the coverage as a corresponding RNA.

We also used HISAT2 (option: -f --dta --rna-strandness F) to identify sequences that did spliced RNA, and STAR (option: --estimate-rspd --append-names --seed-length 15 --strandedness forward) to align the transcript sequence.

In addition, bowtie (option: -f -l 15 --norc -v 1 -k 100) was used to match the repeat sequence in the genome.

Table 19 shows the categories of smRNAs present in *Mus musculus* in RNAcentral.

Table 20 shows the priority of the small RNA category in this study.

Table 19. RNA categoties in RNAcentral Database for species

Taxon ID	Species	Small RNA category
10090	Mus musculus	rRNA, tRNA, piRNA, snoRNA, snRNA, siRNA, Y_RNA, scRNA, vault_RNA

Table 20. Priority of small RNA category

Priority	Small RNA category
1	Known miRNA
2	Novel miRNA
3	piRNA
4	snoRNA
5	snRNA
6	rRNA
7	tRNA
8	siRNA
9	Y RNA
10	scRNA
11	vault RNA
12	Genome
13	Repeat
14	Mycoplasma
15	TruSeqIndexedAdapter

Figure 9 is the bar plot of RNA composition without priority for Control_Evs.
Figure 10 is the pie chart of RNA composition with priority for Control_Evs.
The plots of other samples are located in result_smRNA_excel/RNA_Composition_result/

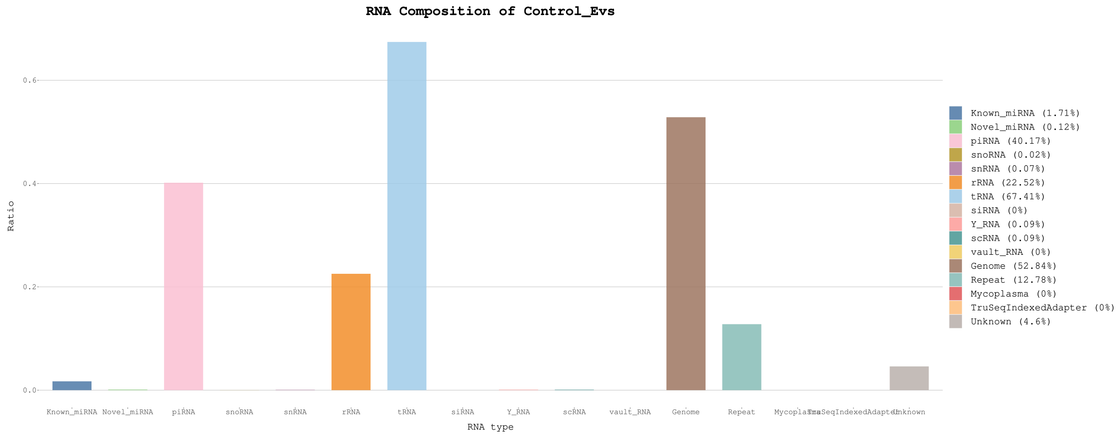

Figure 9. RNA composition without priority for Control_Evs

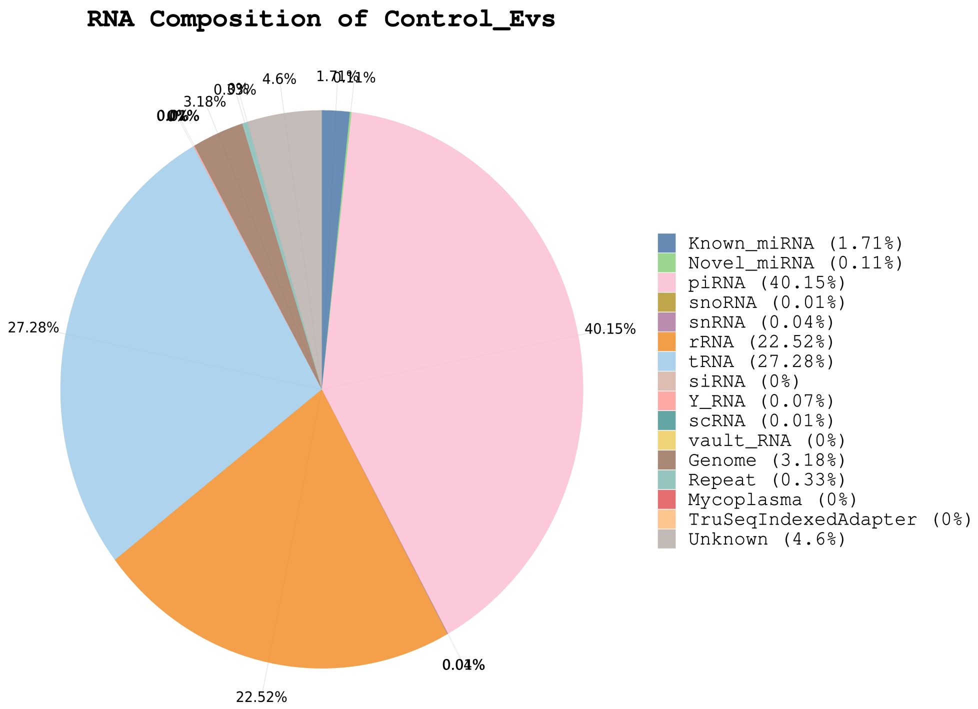

Figure 10. RNA composition with priority for Control_Evs

5. Data Download Information

5. 1. Raw Data

Raw data is the FASTQ file that isn't trimmed adapter sequence.

Download link	File size	md5sum
Control_Evs_1.fastq.gz	1.06G	7cd7d582fa628bdc952fc4fe1d554f45
Control_Evs_2.fastq.gz	861.46M	001dbf89d8dab858f16ce98da3252835
High_Hz_Evs_1.fastq.gz	1.02G	f9fd4bde32fcae7c64966a014152c6da
High_Hz_Evs_2.fastq.gz	889.59M	b16910a9ed1f105046b31c2af3a32b01
Low_Hz_Evs_1.fastq.gz	826.28M	53afebdece32586bc951c737b8184bad
Low_Hz_Evs_2.fastq.gz	817.83M	869e4f1fd2b9986f80ca49daf2f8628b

- fastq.gz : This is a zip file of raw data used in analysis.
- md5sum : In order to verify the integrity of files, md5sum is used. If the values of md5sum are the same, there is no forgery, modification or omission.

5. 2. Analysis Results

Download link	File size
HN00227484_result_smRNA.zip<br>(md5sum: fd15b3ea956de0013a85fba2ac5d37fd)	13.41M
HN00227484_result_smRNA_excel.zip<br>(md5sum: d2cd0efdafbbf3e08e5425ed698aa7b2)	19.32M
HN00227484_processedFASTQ.zip<br>(md5sum: a794235fdedff5b9d92d840582783c31)	1.05G

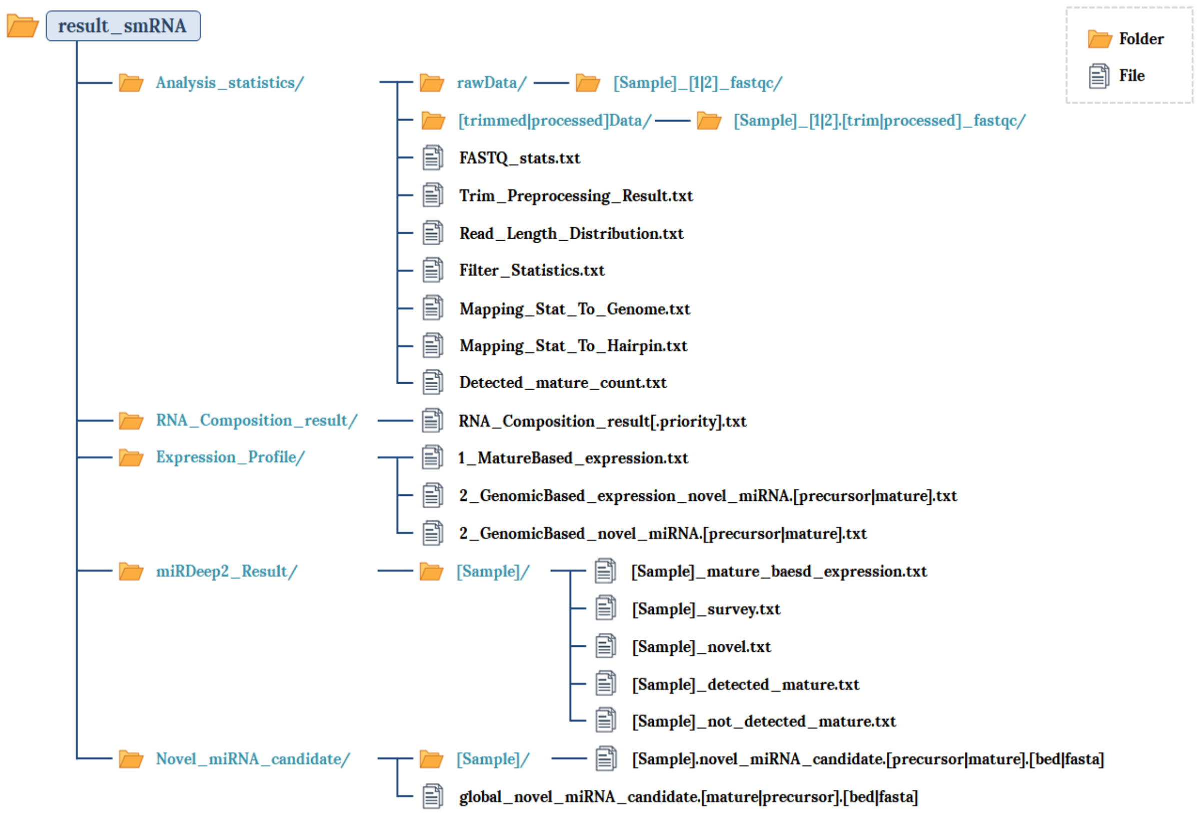
result_smRNA
Folder
File
Analysis_statistics/
rawData/
[Sample]_[1|2]_fastqc/
[trimmed|processed]Data/
[Sample]_[1|2].[trim|processed]_fastqc/
FASTQ_stats.txt
Trim_Preprocessing_Result.txt
Read_Length_Distribution.txt
Filter_Statistics.txt
Mapping_Stat_To_Genome.txt
Mapping_Stat_To_Hairpin.txt
Detected_mature_count.txt
RNA_Composition_result/
RNA_Composition_result[.priority].txt
Expression_Profile/
1_MatureBased_expression.txt
2_GenomicBased_expression_novel_miRNA.[precursor|mature].txt
2_GenomicBased_novel_miRNA.[precursor|mature].txt
miRDeep2_Result/
[Sample]/
[Sample]_mature_baesd_expression.txt
[Sample]_survey.txt
[Sample]_novel.txt
[Sample]_detected_mature.txt
[Sample]_not_detected_mature.txt
Novel_miRNA_candidate/
[Sample]/
[Sample].novel_miRNA_candidate.[precursor|mature].[bed|fasta]
global_novel_miRNA_candidate.[mature|precursor].[bed|fasta]

- result_smRNA_excel
  - Analysis_statistics/
    - ReadLengthDistribution_sample/
      - ReadLengthDistribution_[Sample].png
    - TrimSummary.png
    - ReadLengthDistribution.png
    - ReadLengthDistribution_total.png
    - FilterSummary.png
  - RNA_Composition_result/
    - RNA_Composition_result.priority.png
    - RNA_Composition_result[.priority].[Sample].png
  - Expression_Profile/
    - Expression_Profile.[tri_code].[Ref].miRNA.xlsx
  - miRDeep2_Result/
    - [Sample]/
      - [Sample]_pdf_files/
        - [ProvisionalID].pdf
      - [Sample]_MatureBased_result.xlsx
      - [Sample]_GenomeBased_result.xlsx
  - DEmiRNA_result (Optional)
    - Data Quality Check/
    - Cluster image/
    - Plots/
    - ...
    - data2.xlsx
    - data3_[filtering criteria].xlsx
    - data3_[comparison pair]_[filtering criteria].xlsx

Folder
File

- processedFASTQ
  - [Sample].processed.fastq

Folder
File

Your data will be retained in our server for 3 months.
Should you wish to extend the retention period, please contact us.

6. Appendix

6. 1. Phred Quality Score Chart

Phred quality score numerically express the accuracy of each nucleotide. Higher Q number signifies higher accuracy. For example, if Phred assigns a quality score of 30 to a base, the chances of having base call error are 1 in 1000.

Quality of phred score	Probability of incorrect base call	Base call accuracy	Characters
10	1 in 10	90%	!"#$%&'()*+
20	1 in 100	99%	,-./012345
30	1 in 1000	99.9%	6789:;h=i?
40	1 in 10000	99.99%	@ABCDEFGHIJ

Phred Quality Score Q is calculated with $-10\log_{10}P$, where P is probability of erroneous base call.

6. 2. Programs used in Analysis

6. 2. 1. FastQC v0.11.7

LINK http://www.bioinformatics.babraham.ac.uk/projects/fastqc/

FastQC is a program that performs quality check on the raw sequences before analysis to make sure data integrity. The main function is importing BAM, SAM, FastQ files and providing quick overview on which section has problems. It provides such results as graphs and tables in html files.

6. 2. 2. Cutadapt 4.4

LINK https://cutadapt.readthedocs.org/en/stable/

Cutadapt finds and removes adapter sequences, primers, poly-A tails and other types of unwanted sequence from your high-throughput sequencing reads.

- -q: Try to trim low-quality ends from reads before adapter removal.
- -g: Sequence of an adapter that was ligated to the 5' end.
- -a: Sequence of an adapter that was ligated to the 3' end.
- -O: Minimum overlap length.
- --max-n: The max proportion of N's allowed in a read.
- -m: Discard trimmed reads that are shorter than LENGTH.
- -n: Try to remove adapters at most COUNT times.

6. 2. 3. miRDeep2 2.0.0.8, Bowtie 1.1.2

LINK https://www.mdc-berlin.de/content/mirdeep2-documentation

miRDeep2 is a completely overhauled tool which discovers microRNA genes by analyzing sequenced RNAs. The tool reports known and hundreds of novel microRNAs with high accuracy in seven species representing the major animal clades. The low consumption of time and memory combined with user-friendly interactive graphic output makes miRDeep2 accessible for straightforward application in current reasearch.

6. 2. 4. Bowtie 1.1.2

LINK http://bowtie-bio.sourceforge.net/index.shtml

Bowtie is an ultrafast, memory-efficient short read aligner. Bowtie's speed and small memory footprint are due chiefly to its use of the Burrows-Wheeler index in combination with the novel, quality-aware, backtracking algorithm.

6. 2. 5. HISAT2 version 2.1.0, Bowtie2 2.5.1

LINK https://ccb.jhu.edu/software/hisat2/index.shtml

HISAT2 is a fast and sensitive alignment program for mapping next-generation sequencing reads to genomes. Its first implementation based on an extension of BWT for graphs, designed a graph FM index (GFM). In addition to using one global GFM index, HISAT2 uses a large set of small GFM indexes that collectively cover the whole genome (each index representing a genomic region of 56 Kbp, with 55,000 indexes needed to cover the human population). These small indexes (called local indexes), combined with several alignment strategies, enable rapid and accurate alignment of sequencing reads. This new indexing scheme is called a Hierarchical Graph FM index (HGFM).

6. 2. 6. RSEM verion v1.3.1, STAR 2.6.0c

LINK http://deweylab.github.io/RSEM/

RSEM is a software package for estimating gene and isoform expression levels from RNA-Seq data. The RSEM package provides an user-friendly interface, supports threads for parallel computation of the EM algorithm, single-end and paired-end read data, quality scores, variable-length reads and RSPD estimation. In addition, it provides posterior mean and 95% credibility interval estimates for expression levels.

6. 2. 7. miRBase release 22.1

LINK http://www.mirbase.org/

The miRBase database is a searchable database of published miRNA sequences and annotation. Each entry in the miRBase Sequence database represents a predicted hairpin portion of a miRNA transcript (termed mir in the database), with information on the location and sequence of the mature miRNA sequence (termed miR). Both hairpin and mature sequences are available for searching and browsing, and entries can also be retrieved by name, keyword, references and annotation.

6. 2. 8. RNAcentral version 14.0

LINK https://rnacentral.org/

RNAcentral is a free, public resource that offers integrated access to a comprehensive and up-to-date set of non-coding RNA sequences provided by a collaborating group of Expert Databases representing a broad range of organisms and RNA types.

6. 3. References

1. MARTIN, Marcel. Cutadapt removes adapter sequences from high-throughput sequencing reads. EMBnet. journal, 2011, 17.1: pp. 10-12.
2. FRIEDLAENDER, Marc R., et al. Discovering microRNAs from deep sequencing data using miRDeep. Nature biotechnology, 2008, 26.4: 407.
3. FRIEDLAENDER, Marc R., et al. miRDeep2 accurately identifies known and hundreds of novel microRNA genes in seven animal clades. Nucleic acids research, 2011, 40.1: 37-52.
4. LANGMEAD, Ben, et al. Ultrafast and memory-efficient alignment of short DNA sequences to the human genome. Genome Biol, 2009, 10.3: R25.
5. KIM, Daehwan; LANGMEAD, Ben; SALZBERG, Steven L. HISAT: a fast spliced aligner with low memory requirements. Nature methods, 2015, 12.4: 357-360.
6. LANGMEAD, Ben; SALZBERG, Steven L. Fast gapped-read alignment with Bowtie 2. Nature methods, 2012, 9.4: 357.
7. LI, Bo; DEWEY, Colin N. RSEM: accurate transcript quantification from RNA-Seq data with or without a reference genome. BMC bioinformatics, 2011, 12.1: 1.
8. DOBIN, Alexander, et al. STAR: ultrafast universal RNA-seq aligner. Bioinformatics, 2013, 29.1: 15-21.
9. KOZOMARA, Ana; GRIFFITHS-JONES, Sam. miRBase: annotating high confidence microRNAs using deep sequencing data. Nucleic acids research, 2013, 42.D1: D68-D73.
10. RNACENTRAL CONSORTIUM. RNAcentral: a comprehensive database of non-coding RNA sequences. Nucleic acids research, 2016, gkw1008.
11. LI, Heng, et al. The sequence alignment/map format and SAMtools. Bioinformatics, 2009, 25.16: 2078-2079.
12. QUINLAN, Aaron R.; HALL, Ira M. BEDTools: a flexible suite of utilities for comparing genomic features. Bioinformatics, 2010, 26.6: 841-842.

Research use only

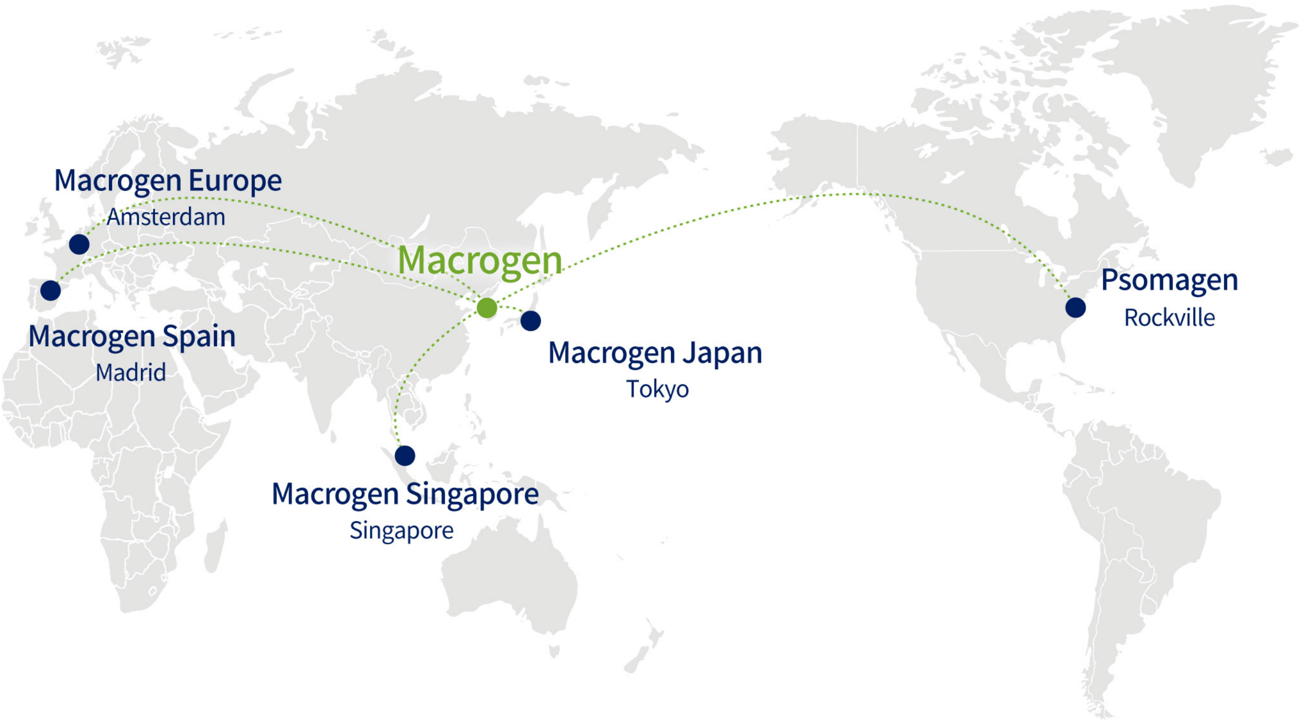

HEADQUARTER

Macrogen, Inc.

Laboratory, IT and Business Headquarter & Support Center

[08511] 1001, 10F, 254, Beotkkot-ro, Geumcheon-gu, Seoul, Republic of Korea (Gasan-dong, World Meridian 1)
Tel: +82-2-2180-7000
Email1: ngs@macrogen.com(Overseas)
Email2: ngskr@macrogen.com (Republic of Korea)
Web: www.macrogen.com
LIMS: dna.macrogen.com

SUBSIDIARY

Macrogen Europe

Laboratory, Business & Support Center

Meibergdreef 57, 1105 BA, Amsterdam, the Netherlands
Tel: +31-20-333-7563
Email: ngs@macrogen.eu

Macrogen Singapore

Laboratory, Business & Support Center

3 Biopolis Drive #05-18, Synapse, Singapore 138623
Tel: +65-6339-0927
Email: info-sg@macrogen.com

Psomagen (Macrogen USA)

Laboratory, Business & Support Center

1330 Piccard Drive, Suite 103, Rockville, MD 20850, United States
Tel: +1-301-251-1007
Email: inquiry@psomagen.com

Macrogen Japan

Laboratory, Business & Support Center

16F Time24 Building, 2-4-32 Aomi, Koto-ku, Tokyo 135-0064 JAPAN
Tel: +81-3-5962-1124
Email: ngs@macrogen-japan.co.jp

BRANCH

Macrogen Spain

Laboratory, Business & Support Center

Av. Sur del Aeropuerto de Barajas, 28. Office B-2, 28042 Madrid, Spain
Tel: +34-911-138-378
Email: info-spain@macrogen.com

Humanizing Genomics
macrogen
