# Supplementary material for: Small Extracellular Vesicle Release Following Electrical Pulse Stimulation of C2C12 Myotubes: Effects on microRNA Cargo and Myoblast Migration and Differentiation
Source: Int J Mol Sci. 2026 May 12;27(10):4320. doi: 10.3390/ijms27104320 (PMC13206931; doi:10.3390/ijms27104320)
Supplement: Supplementary file 1 [file ijms-27-04320-s001.zip › ijms-4231158-supplementary/Supplementary file 5 - R Studio script 54.pdf]

```
# --- 1. INSTALLATION & LOADING ---
if (!requireNamespace("BiocManager", quietly = TRUE)) install.packages("BiocManager")
pkgs <- c("readxl", "writexl", "dplyr", "tidyr", "edgeR", "multiMiR", "clusterProfiler",
          "org.Mm.eg.db", "janitor", "tibble", "ggplot2", "enrichplot", "patchwork",
          "RColorBrewer", "reshape2", "scales", "stringr")

for (p in pkgs) {
  if (!require(p, character.only = TRUE)) {
    if (p %in% c("multiMiR", "clusterProfiler", "org.Mm.eg.db", "edgeR")) {
      BiocManager::install(p, update = FALSE, ask = FALSE)
    } else { install.packages(p) }
    library(p, character.only = TRUE)
  }
}

# --- 2. DATA LOADING & TMM NORMALIZATION ---
read_miRNA <- function(file_path, name) {
  df <- read_excel(paste0(file_path, ".xlsx"))
  df_clean <- df %>%
    dplyr::select(miRNA_ID = miRBase_Precursor_ID, Raw_Counts = Total_Read_Count) %>%
    dplyr::filter(!is.na(miRNA_ID)) %>%
    dplyr::group_by(miRNA_ID) %>%
    dplyr::summarise(Raw_Counts = sum(Raw_Counts, na.rm = TRUE), .groups = "drop") %>%
    dplyr::rename(!!paste0(name, "_Raw_Reads") := Raw_Counts)
  return(df_clean)
}

control_df <- read_miRNA("Control", "Control")
high_df    <- read_miRNA("High", "High")
low_df     <- read_miRNA("Low", "Low")
```

```
raw_counts <- control_df %>%
  full_join(high_df, by = "miRNA_ID") %>%
  full_join(low_df, by = "miRNA_ID") %>%
  mutate(across(where(is.numeric), ~replace_na(., 0))) %>%
  column_to_rownames("miRNA_ID")

dge  <- DGEList(counts = raw_counts)
keep <- rowSums(cpm(dge) > 15) >= 1
dge  <- dge[keep, , keep.lib.sizes = FALSE]
dge  <- calcNormFactors(dge, method = "TMM")
norm_cpm <- as.data.frame(cpm(dge, normalized.lib.sizes = TRUE)) %>%
  rename_with(~gsub("_Raw_Reads", "_TMM_CPM", .x))

master_summary <- as.data.frame(dge$counts) %>%
  rownames_to_column("miRNA_ID") %>%
  left_join(rownames_to_column(norm_cpm, "miRNA_ID"), by = "miRNA_ID") %>%
  mutate(
    High_vs_Control_Log2FC = log2((High_TMM_CPM + 1) / (Control_TMM_CPM + 1)),
    Low_vs_Control_Log2FC  = log2((Low_TMM_CPM + 1) / (Control_TMM_CPM + 1)),
    High_vs_Low_Log2FC     = log2((High_TMM_CPM + 1) / (Low_TMM_CPM + 1))
  )

write_xlsx(master_summary, "Master_miRNA_TMM_Log2FC_Results.xlsx")

# --- 3. CORE ANALYSIS & PLOTTING FUNCTION ---
run_muscle_analysis <- function(sig_mirs, comparison_name, file_suffix) {
  if (length(sig_mirs) < 3) return(NULL)

  res <- tryCatch({
    suppressWarnings(get_multimir(mirna = sig_mirs, org = "mmu", table = "all", summary = TRUE))
  }, error = function(e) return(NULL))
```

```
  if (is.null(res)) return(NULL)

  target_data <- as_tibble(lapply(as.data.frame(res@data), function(x) as.vector(x))) %>%
    dplyr::filter(database %in% c("mirdb", "tarbase", "mirtarbase")) %>%
    dplyr::select(mature_mirna_id, target_symbol, target_entrez) %>%
    distinct()

  ego <- tryCatch({
    enrichGO(gene = unique(as.character(target_data$target_entrez)), OrgDb = org.Mm.eg.db, ont =
"BP", pvalueCutoff = 0.5, readable = TRUE)
  }, error = function(e) return(NULL))

  if(is.null(ego)) return(NULL)

  muscle_terms <- "satellite cell|myoblast|muscle|myogenic|myotube|sarcomere|actin
filament|fusion|myogenesis|skeletal.*muscle|myoblast differentiation|myoblast
proliferation|myoblast migration|satellite cell activation|satellite cell migration|satellite cell
proliferation"

  ego_muscle <- as.data.frame(ego) %>%
    dplyr::filter(grepl(muscle_terms, Description, ignore.case = TRUE)) %>%
    mutate(GeneRatioNumeric = sapply(GeneRatio, function(x) {
      parts <- as.numeric(unlist(strsplit(x, "/")))
      return(parts[1] / parts[2])
    }))

  if(nrow(ego_muscle) == 0) return(NULL)
  write_xlsx(ego_muscle, paste0("Muscle_Pathways_", file_suffix, ".xlsx"))

  annotate_stat_cascade <- function(desc) {
    families <- c("Wnt", "MAPK", "Notch", "TGF-beta", "PI3K-Akt", "mTOR", "JAK-STAT", "Hedgehog",
"BMP", "FGF")
```

```
    found <- families[sapply(families, function(x) grepl(x, desc, ignore.case = TRUE))]
    label <- desc
    if(length(found) > 0) label <- paste0(desc, "\n[", paste(found, collapse=", "), "]")
    return(label)
  }

  ego_muscle$AnnotatedDesc <- sapply(ego_muscle$Description, function(x)
str_wrap(annotate_stat_cascade(x), width = 40))
  plot_data <- ego_muscle[1:min(15, nrow(ego_muscle)), ]

  # --- PLOT 1: GO DOTPLOT ---
  p1 <- ggplot(plot_data, aes(x = GeneRatioNumeric, y = reorder(AnnotatedDesc,
GeneRatioNumeric))) +
    geom_point(aes(size = Count, fill = p.adjust), shape = 21, color = "black", stroke = 1.3) +
    # FIXED GLOBAL P-ADJUST SCALE
    scale_fill_gradientn(
      colors = rev(brewer.pal(11, "PuOr")),
      trans = "log10",
      limits = c(0.0001, 0.05), # Hard-coded global range
      breaks = c(0.0001, 0.001, 0.01, 0.05),
      labels = scientific_format(),
      oob = scales::squish,     # Keep values outside range visible at the extremes
      guide = guide_colorbar(order = 1, barheight = unit(10, "cm"), title = "p.adjust")
    ) +
    scale_size_area(max_size = 24) +
    coord_cartesian(xlim = c(0, 0.06)) +
    scale_x_continuous(breaks = seq(0, 0.06, by = 0.01)) +
    theme_minimal() +
    labs(title = paste0(comparison_name), y = "Signaling Process", x = "Gene Ratio") +
    theme(
      plot.title = element_text(size = 36, face = "bold", hjust = 0.5),
      axis.text.y = element_text(size = 24, face = "bold", lineheight = 0.9),
```

```r
    axis.text.x = element_text(size = 24, face = "bold"),
    axis.title = element_text(size = 28, face = "bold"),
    legend.position = "right", legend.box = "vertical", legend.key.size = unit(2.5, "cm"),
    legend.title = element_text(size = 22, face = "bold"), legend.text = element_text(size = 20)
  )

# --- PLOT 2: TOP TARGETS BAR PLOT ---
all_genes <- unique(unlist(strsplit(plot_data$geneID, "/")))
top_t <- target_data %>% dplyr::filter(target_symbol %in% all_genes) %>%
  dplyr::count(target_symbol) %>% dplyr::arrange(desc(n)) %>% head(20)

top_t$label <- sapply(top_t$target_symbol, function(s) {
  m <- ego_muscle$Description[grepl(s, ego_muscle$geneID)][1]
  full_lab <- paste0(s, "\n(", annotate_stat_cascade(m), ")")
  return(str_wrap(full_lab, width = 45))
})

p2 <- ggplot(top_t, aes(x = reorder(label, n), y = n)) +
  geom_bar(stat = "identity", fill = "#d95f02", width = 0.75) +
  geom_text(aes(label = sprintf("%.0f", as.numeric(n))), hjust = -0.5, size = 12, fontface = "bold") +
  coord_flip(ylim = c(0, 8)) +
  scale_y_continuous(breaks = seq(0, 8, by = 1)) +
  theme_minimal() +
  labs(title = "Top Muscle Targets", x = "", y = "Target Frequency") +
  theme(
    plot.title = element_text(size = 36, face = "bold", hjust = 0.5),
    axis.text.y = element_text(size = 22, face = "bold", lineheight = 0.95),
    axis.text.x = element_text(size = 24, face = "bold"),
    axis.title = element_text(size = 28, face = "bold")
  )
```

```
  combined <- (p1 + p2) + plot_layout(widths = c(1, 1))

  ggsave(paste0("Figure_Muscle_", file_suffix, ".png"), combined, width = 48, height = 25, units = 
"in", dpi = 300, limitsize = FALSE)

}


# --- 4. EXECUTION LOOP (6 COMPARISONS) ---

comps <- list(

  "High_vs_Control_UP"   = list(f = master_summary$High_vs_Control_Log2FC > 1,   n = "High vs 
Control UP",   s = "High_vs_Control_UP"),

  "High_vs_Control_DOWN" = list(f = master_summary$High_vs_Control_Log2FC < -1,  n = "High vs 
Control DOWN", s = "High_vs_Control_DOWN"),

  "Low_vs_Control_UP"    = list(f = master_summary$Low_vs_Control_Log2FC > 1,    n = "Low vs 
Control UP",    s = "Low_vs_Control_UP"),

  "Low_vs_Control_DOWN"  = list(f = master_summary$Low_vs_Control_Log2FC < -1,   n = "Low vs 
Control DOWN",  s = "Low_vs_Control_DOWN"),

  "High_vs_Low_UP"       = list(f = master_summary$High_vs_Low_Log2FC > 1,       n = "High vs Low 
UP",       s = "High_vs_Low_UP"),

  "High_vs_Low_DOWN"     = list(f = master_summary$High_vs_Low_Log2FC < -1,      n = "High vs Low 
DOWN",     s = "High_vs_Low_DOWN")

)


for (n in names(comps)) {

  try(run_muscle_analysis(master_summary$miRNA_ID[comps[[n]]$f], comps[[n]]$n, comps[[n]]$s))

}
```
