# Supplementary figures and images for: Small Extracellular Vesicle Release Following Electrical Pulse Stimulation of C2C12 Myotubes: Effects on microRNA Cargo and Myoblast Migration and Differentiation
Source: Int J Mol Sci. 2026 May 12;27(10):4320. doi: 10.3390/ijms27104320 (PMC13206931; doi:10.3390/ijms27104320)

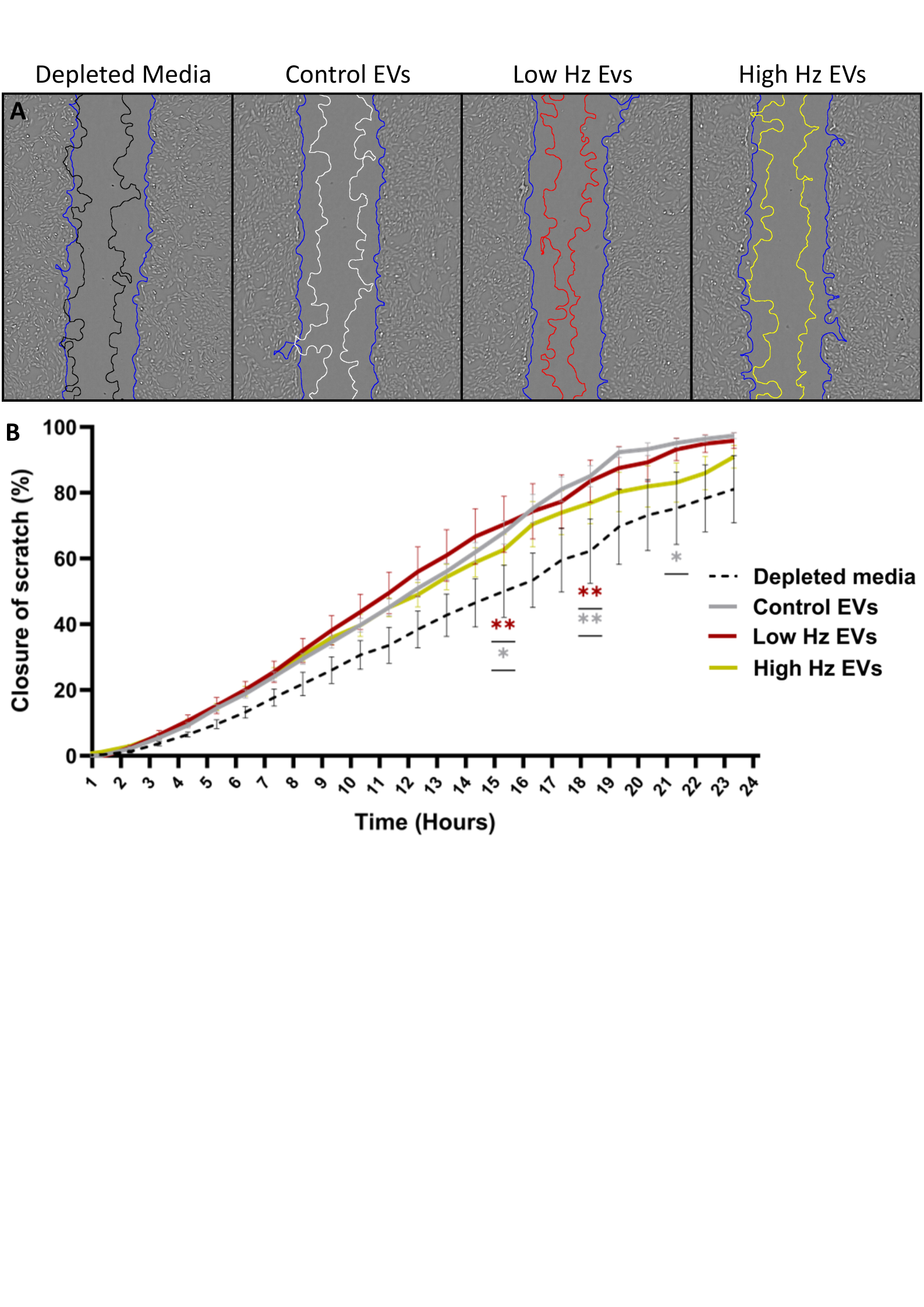

Supplement: Supplementary file 1 [file ijms-27-04320-s001.zip › ijms-4231158-supplementary/Supplementary file 2 - Figure 7 with depleted media control.png]
